# Use of routine health information systems to monitor disruptions of coverage of maternal, newborn, and child health services during COVID-19: A scoping review

**DOI:** 10.7189/jogh.13.06002

**Published:** 2023-02-10

**Authors:** Debra Jackson, Elizabeth Katwan, Claudia Boehm, Theresa Diaz

**Affiliations:** 1 London School of Hygiene and Tropical Medicine, London UK; 2 University of the Western Cape School of Public Health, Cape Town, South Africa; 3 Maternal, Newborn, Child and Adolescent Health and Ageing, World Health Organization, Geneva, Switzerland

## Abstract

**Background:**

The COVID-19 pandemic is a unique global health challenge which disrupted essential health services (EHS). Most early data related to EHS during the COVID-19 pandemic came from country and regional “pulse” surveys conducted by the World Health Organization (WHO) and United Nations Children’s Fund (UNICEEF), which relied on respondent perceptions and not necessarily routine health information system (RHIS) data. By conducting a scoping review, we aimed to describe the use of RHIS data for monitoring changes in EHS coverage for maternal, newborn, and child health (MNCH) during the COVID-19 pandemic.

**Methods:**

We performed a scoping review using Sample, Phenomenon of Interest, Design, Evaluation, Research type (SPIDER) and Preferred Reporting Items for Systematic Reviews and Meta-Analyses – Scoping Review (PRISMA-SCR) guidelines. We included descriptive or analytic reports on the availability and use of RHIS data published in peer-reviewed, pre-publication, or gray literature on MNCH essential health services coverage during the COVID-19 pandemic. The following databases were searched for studies published between January 2020 and May 2022: PubMed/MEDLINE, Google Scholar, Google, MedRXiv (pre-publication), Embase, CINAHL, Cochrane, Campbell, and OpenGrey. A single reviewer screened the titles, abstracts, and full texts of the retrieved publications, while a second reviewer screened 20% of the total sample. Publications were tabulated by WHO Region, World Bank income group, country, data sources, study topic, and period. We used content analysis to qualitatively describe the trends and use of data for policy or programming in the studies.

**Results:**

We included 264 publications after the full-text review. The publications came from 81 countries, covering all WHO regions and World Bank income groups. The most common data sources were hospital information systems (27%) and primary health care management information systems (26%). Most studies examined data trends before COVID-19 compared to periods during COVID-19. Most publications reported a decrease in MNCH services (45%). Reports with follow-up beyond August 2020 (first six months of pandemic) were significantly more likely to report recovery of service coverage (8% vs 30%, *P* < 0.001). Low- and middle-income countries reported significantly higher morbidity and/or mortality in COVID-19 periods than high-income countries (54% vs 30%, *P* < 0.001). Less than 10% of reports described RHIS data quality specifically during the COVID-19 period and only 22% reported program mitigation strategies to address reductions noted from routine data.

**Conclusion:**

Results suggest awareness and usefulness of RHIS to monitor MNCH service disruptions during the COVID-19 pandemic. However, with only 22% of reports including descriptions of policy or program adaptations, use of RHIS data to monitor MNCH service disruptions was not necessarily followed by data-informed policies or program adaptations. RHIS data on MNCH services should be strengthened to enable its use by program managers and policymakers to respond to direct and indirect effects of future public health emergencies.

**Registration:**

Open Science Framework (available at: https://osf.io/usqp3/?view_only=94731785fcba4377adfa1bdf5754998d).

As the COVID-19 pandemic evolved, it became clear that coverage of health services for maternal, newborn, and child health (MNCH) had been disrupted [1,2]. Countries reported more than half of key MNCH services (such as antenatal care and sick child visits) were disrupted in the third quarter of 2020, with fewer disruptions reported in the fourth quarter of 2021, although approximately one third of countries still reported disruptions to these services [1,2], This information on disruptions to EHS during COVID-19 came from country and regional “pulse” surveys which rely on respondent perceptions rather than routine health information system (RHIS) data. RHIS data allow managers to monitor the utilization of health services by collecting and reporting on health service data from health facilities. RHIS collect health service data directly from the health facilities through their health care workers. It provides frequent (e.g. monthly) and/or almost real-time information on service performance and quality at all health system levels, enabling regular progress monitoring and timely identification and solving of problems. RHIS creates an integrated environment for program-specific and cross-cutting data use [3]. Its key components include administrative records systems (e.g. national health accounts), service records systems (e.g. immunizations administered, health management information systems), and individual records systems (e.g. patient medical records), but do not include population-based data sources (e.g. census, vital registration, population-based surveys) [3]. RHIS data serve a wide range of purposes, including patient/client management, facility management, disease surveillance, monitoring of service provision, resource allocation, and policymaking [4].

By conducting a scoping review, we aimed to describe the global availability and use of RHIS data for monitoring changes in delivery of essential MNCH services during the COVID-19 pandemic. Thus, we wanted to describe 1) how RHIS data were used to determine disruption of essential MNCH services during the COVID-19 pandemic, 2) if changes in RHIS data quality and reporting were reported (e.g. timeliness, missing data, data quality) and whether that impacted the interpretation of findings, e.g. reduction in EHS coverage data due to reduced data reporting not necessarily reduced services, and 3) whether the findings on MNCH service disruptions from the analysis of RHIS during COVID-19 resulted in documented policy responses and/or program adaptations and if there were any patterns or trends across countries?

We based the study questions on the Sample, Phenomenon of Interest, Design, Evaluation, Research type (SPIDER) approach used for qualitative and mixed methods reviews [5].

- Sample: available literature on mothers, newborns, and children in all countries during the COVID-19 pandemic period January 2020 through May 2022.
- Phenomenon of interest: availability and use of routine data to monitor changes or disruptions in coverage of essential health services (EHS) [6] for mothers, newborns, and children during the 2020-2022 COVID-19 pandemic period, including findings and any use of the RHIS data for MNCH programme adjustment.
- Design: research or findings based on analysis or use of RHIS data, primarily including data common in health management information systems (HMIS). Other potential sources could include national health registers or service delivery registers, surveillance systems (e.g. Integrated Disease Surveillance and Response tools (IDSR), community health information systems (CHIS) in use by community health workers, logistics management information systems (LMIS) for commodities, human resource health information systems (HRHIS)), or routine client interviews/satisfaction surveys.
- Evaluation: descriptive or analytic reports published by countries, global/regional initiatives, or researchers in peer-reviewed, pre-publication, or grey literature, or from country-level published databases.
- Research type: quantitative or mixed methods research and reports on routine data use for MNCH services during COVID-19 pandemic 2020-2022.

## METHODS

The methodology followed the scoping literature review process as outlined by Tawfik et al. [7], Siddaway et al. [8]. We followed the 2018 Preferred Reporting Items for Systematic Reviews and Meta-Analyses – Scoping Review (PRISMA-SCR) guidelines [9] in reporting the study.

### Protocol registration

We registered the study protocol at the Open Science Framework: https://osf.io/usqp3/?view_only=94731785fcba4377adfa1bdf5754998d.

### Eligibility criteria

We included descriptive or analytic reports on the availability, use, and findings from RHIS data published by countries, global/regional initiatives, or researchers in peer-reviewed, pre-publication, or gray literature, or from country-level published databases on MNCH EHS during the COVID-19 pandemic (between January 2020 and May 2022) and policy responses to mitigate the impact of COVID-19 on MNCH EHS.

We excluded clinical trials, reports on COVID-19 testing or vaccine development programmes, COVID-19 specific treatment in MNC populations (e.g. clinical treatment protocols), modeling exercises, commentaries without comparative data, protocols, program adjustment reports without comparative data, studies with populations other than MNC, non-routine data (e.g. specially collected research data), surveys, and studies published in a language other than English. We did not include other systematic reviews or scoping reviews, but we checked their reference lists for potentially eligible studies.

### Information sources

We searched PubMed/MEDLINE, Google Scholar, Google, MedRXiv (pre-pub), Embase, CINAHL, Cochrane, Campbell, and OpenGrey. The first author developed the search strategy in consultation with co-authors. An external WHO scoping review expert (who was not a part of the study) reviewed the protocol and search strategy reviewed.

### Search terms

The following terms were used in the database search:

1. Health Services or Essential Health Services
2. Maternal or Antenatal or Perinatal or Postnatal or Newborn or Neonatal or Child* or Adolescen*
3. Corona* or COVID* or SARS-COV-2
4. RHIS or Routine Health Information Systems or Routine Data or Health Information Systems or Health management information systems
5. Other possible MNCH terms: IMCI or iCCM or DTP3 or ARI

These terms were combined using Boolean operator as follows:

- 1 + 2 + 3 + 4
- 1 + 2 + 3 + 5
- 1 (Essential Health Services) + 3 (COVID*).

The following is an example of a search string we used in the MEDLINE database: (Health Services or Essential Health Services) and (Maternal or Antenatal or Perinatal or Postnatal or Newborn or Neonatal or Child* or Adolescen*) and (Corona* or COVID* or SARS-COV-2) and (RHIS or Routine Health Information Systems or Routine Data or Health Information Systems or Health management information systems)

### Selection of sources of evidence

The first author searched the databases for studies published between January 2020 and May 17, 2022. All retrieved results were imported into the Zotero reference manager Zotero [10]. After deduplication, a single independent reviewer (DJ) first screened the titles, followed by the abstracts and full texts of the remaining sources. Studies were selected based on adherence to above-mentioned selection criteria. An independent reviewer (CB) reviewed 20% of included and 20% of excluded reports to assure methodologic agreement. Queries and discrepancies from the independent reviewer were discussed with all authors until a consensus was achieved. No major adjustments were needed after independent review.

### Data charting process and data items

The first author extracted the data from all included studies into a Microsoft Excel [11] database using a pre-developed data extraction tool, which was reviewed and refined by all co-authors after the initial 20 reviews, with ongoing discussion by all authors during the review period to assure consensus. The extracted data are described in **Box 1**, which shows the quantitative categories according to which most data were extracted. We extracted the described data quality assurance, data trends for MNCH outcomes (MNCH morbidity and/or mortality were used as a combined outcome variable), data used for program adjustments/ mitigation, and outcome/impact of program adjustment as qualitative text directly from manuscripts.

Box 1 **Data extraction items**

- Authors
- Title
- Citation
- MOH or manager authors (yes/no)
- Reference source (peer-reviewed literature, grey literature, country database, pre-publication literature)
- World Health Organization Region (WHO Regions AFRO, AMRO, EMRO, EURO, SEARO, WPRO). Available: https://www.who.int/about/who-we-are/regional-offices. Accessed: 18 August 2022.
- World Bank country classifications by income level 2022-2033 (HIC or LMIC). Available: https://blogs.worldbank.org/opendata/new-world-bank-country-classifications-income-level-2022-2023. Accessed: 18 August 2022]
- Country/countries included in the report
- Populations covered (Maternal, Neonatal, Children (0-18))
- Topic(s) (child abuse, child health, EMS/hospitalization, HIV/tb, immunization, maternal health, maternal & child health, maternal & newborn health, mental health, newborn health, specialty services
- RHIS type (admin/billing, national statistics office, EIR, EMR, electronic primary health care management information system (e.g., DHIS2), HMIS (including emergency medical systems), Logistics management information systems, network routine database, paper data (HMIS or EMR), registry data)
- Data quality assurance described
- Data period covered
- Follow-up beyond August 2020
- Data trends for MNCH service coverage (decrease, decreased with recovery, mixed, stable)
- Data trends for MNCH outcomes (MNCH morbidity and/or mortality were used as a combined outcome variable).
- Data used for programme adjustments/ mitigation
- Mitigation category
- Outcome/impact of programme adjustment

### Critical appraisal of sources of evidence

Quality appraisal of included publications was not a focus in this review, as we focused primarily on descriptive reports. We therefore did not include risks of bias or conflicts of interest.

### Synthesis of results

Following data extraction, we conducted quantitative and qualitative/narrative syntheses [12]. We tabulated the studies [12] by WHO Region, World Bank income group, country, data sources, study topic, and period. We used content analysis [12] qualitatively describe the trends and use of data for policy or programming. We generated ross-country lessons and developed recommendations for monitoring and addressing MNCH EHS in future emergencies in consultation with the WHO. Statistical analyses were done in Microsoft Excel [11].

## RESULTS

### Selection of Sources of Evidence

**Figure 1:**
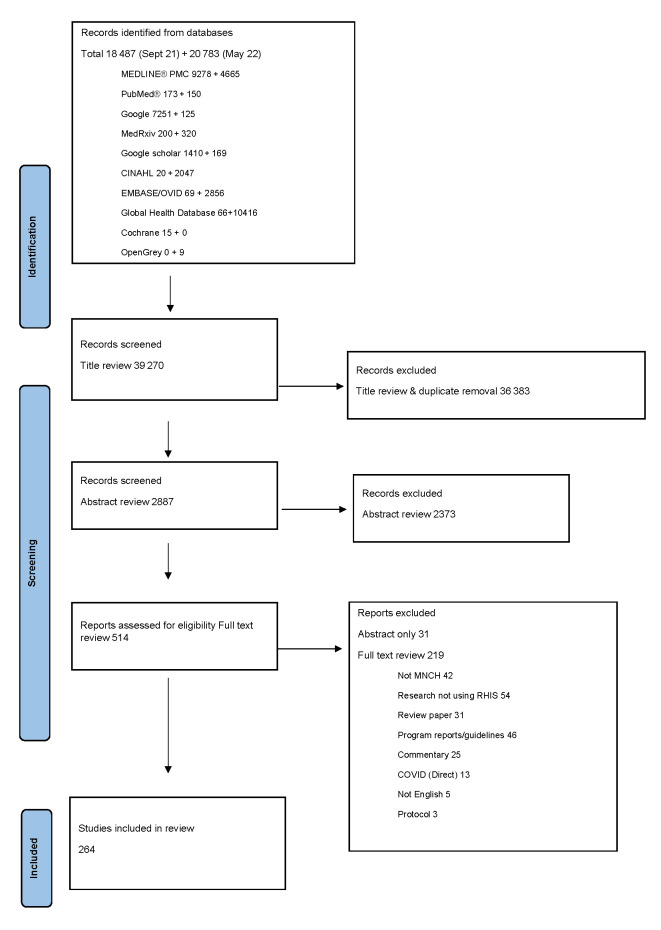
PRISMA-SCR 2020 flow diagram. MNCH – maternal, newborn, and child health, RHIS – routine health information system. Source Page MJ, McKenzie JE, Bossuyt PM, Boutron I, Hoffmann TC, Mulrow CD, et al. The PRISMA-SCR 2020 statement: an updated guideline for reporting scoping reviews. BMJ 2021;372:n71. doi: 10.1136/bmj.n71. For more information, visit: http://www.PRISMA-ScR-statement.org/

We identified a total of 39 248 studies from the selected databases (**Figure 1**), with an additional 22 identified during reference screening of review articles or reference lists from full text reports, for a total of 39 270 studies. After deduplication and title review, 2887 studies remained. After the abstract screening, 514 studies were left; 483 had full texts available and 31 had only abstracts (primarily from published scientific presentations). During the full-text review, 219 studies were excluded for the following reasons: not MNCH (n = 42), research not using RHIS (n = 54), review paper (n = 31), program reports/guidelines (n = 46), commentary (n = 25), COVID-19 (direct) (n = 13), not in English language (n = 5), protocol (n = 3). After full text review, we included 264 reports. The full data are presented in Table S1 in the **Online Supplementary Document**.

### Characteristics of sources of evidence

**Table 1** summarizes the characteristics of included studies. The 264 reports came from 81 countries, primarily from WHO regions for Africa, Europe, and the Americas, with fewer studies from Eastern Mediterranean, South-East Asian, and Western Pacific regions. For World Bank country classifications by income level, 115 (45%) were from low- and middle-income countries (LMICs) and 156 (59%) were from high income countries (HICs). Most reports (n = 245, 92%) were from peer reviewed literature; only 19 (7%) were from gray literature and pre-publication reports.

**Table 1:** Characteristics of included reports (n = 264)

Characteristic	N (%)
**Ministry of Health or program manager co-authors**	
Yes	136 (52%)
No	128 (48%)
WHO region*	
Africa	72 (27%)
Americas	80 (30%)
Eastern Mediterranean	20 (8%)
Europe	69 (26%)
South-East Asia	33 (13%)
Western Pacific	24 (9%)
**World Bank income group†**	
High income countries	156 (59%)
Low- and middle-income countries	115 (45%)
**Reference source**	
Peer review	245 (92%)
Grey literature/country database	7 (3%)
Pre-publication	12 (4%)

*WHO Region >264 as multiple paper covered >1 region.

**†**The World Bank Income group contains >264 records, as three papers included both low- and middle-income and high-income countries.

The most common RHIS type (**Table 2**) were hospital information systems (27%) and primary health care (PHC) management information systems (e.g. District Health Information System 2 (DHIS2)) (26%). The majority looked at children (62%), followed by pregnant, intrapartum, or postpartum women (32%), and newborns (12%). Common topics investigated included child health (e.g. well and sick child visits, 34%), maternal

**Table 2:** Data sources, populations, and topics of included reports (n = 264)

Characteristic	N
Data source – RHIS type*	
Admin/billing	13 (5%)
EIR	19 (7%)
EMR	40 (15%)
PHC health management information system	68 (26%)
Hospital management information system (incl. EMS)	71 (27%)
Network database (e.g. across hospitals or systems)	11 (4%)
Paper-based HMIS/MR	26 (10%)
Registry/surveillance	19 (7%)
**Population**	
Children	163 (62%)
Maternal	29 (11%)
Maternal and children	40 (15%)
Maternal and neonatal	16 (6%)
Neonatal	16 (6%)
**Topic**	
Child abuse	7 (3%)
Child health	24 (9%)
Child specialty services	22 (8%)
EMS/hospitalization	59 (22%)
HIV/TB	8 (3%)
Immunization	40 (15%)
Maternal health	20 (8%)
Maternal and child health	38 (14%)
Maternal and newborn health	15 (6%)
Mental health	9 (3%)
Newborn health	32 (12%)
Discussed data quality/DQA	45 (17%)
Discussed data quality specific to COVID-19 period	23 (9%)
Discussed program adjustments/mitigation	58 (22%)
RHIS used to monitor or evaluate program adjustments/mitigation	48 (18%)
Follow trends beyond August 2020	112 (42%)

RHIS – routine health information system, EIR – electronic immunization register, EMR – electronic medical record, HIV – human immunodeficiency virus, TB – tuberculosis, EMS – emergency medical service, DQA -

*RHIS type >264 as three papers used two data sources; all sources listed were digital except where “paper-based” is specified.

### Results of individual sources of evidence

#### Description of service disruptions

Most reports were before and during/after studies comparing pre-COVID-19 to during/post-COVID-19 periods. Two studies used during COVID-19 time periods compared to modeled expected values based on prior data trends.

#### Disruptions to RHIS quality and reporting

Forty-five (17%) of the included studies discussed data quality issues. Twenty-two (8%) reported generally on data quality issues consistent with usual list of routine data limitations (e.g. missing data, delayed data, outliers) without any specific analyses if these issues changed during the COVID-19 period. Twenty-three (9%) studies discussed potential data quality issues specific to COVID-19 periods (**Table 2**); only 12 (5%) of them attempted to describe the impact on data quality during COVID-19, five (2%) of which found impacts on data quality during COVID-19, while seven (3%) did not find any impact. Masresha et.al. [13] noted a small reduction in the completeness of HMIS reports in South Sudan but not the other four countries in their study, while Shrinivasan et.al. [14] noted increased delayed reporting in India. Amazou et.al. [15], found reporting completeness, and minimal outliers, or missing values in 11 of the countries in their study to be stable, except for Nigeria. Data quality issues during COVID-19 in Nigeria were also confirmed in a report from the Nigeria Ministry of Health [16]. Ayele et al. [17] from Ethiopia found evidence of reduced data quality assurance (DQA) activities during the COVID period, while Mbithi et.al.[18] from Kenya noted increased DQA activities, though neither report if there were any impacts on data.

### Synthesis of results

#### Patterns or trends across countries – coverage and morbidity/mortality

Most publications reported a decrease in MNCH service coverage compared to pre-pandemic levels without a recovery within the study period (45%). A smaller proportion reported initial decreases with later recovery to pre-pandemic levels (17%). Many reports showed mixed results across indicators in the same report (26%), and a small percentage reported stable (8%) or increased (4%) service coverage across compared time periods (**Table 3**).

**Table 3:** Data trends for services coverage, and morbidity/mortality

Data tends	Total	HIC*	LMIC*
**Coverage (n (%))**			
Decreased	106 (45%)	58 (43%)	47 (47%)
Decreased with recovery	42 (17%)	19 (14%)	21 (19%)
Mixed	64 (26%)	41 (31%)	23 (23%)
Stable	20 (8%)	12 (9%)	8 (7%)
Increase	10 (4%)	5 (3%)	4 (4%)
Total	242 (100%)	135 (100%)	103 (100%)
**Morbidity/mortality (n (%))**			
Increased	27 (39%)	13 (30%)	14 (54%)
Mixed	9 (13%)	5 (11%)	4 (15%)
Stable	18 (26%)	13 (30%)	5 (19%)
Decreased	16% (22%)	13 (30%)	3 (12%)
Total	70 (100%)	44 (100%)	26 (100%)

HIC – high-income country, LMIC – low- and middle-income country

*Four papers combined data for HICs and LMICs so could not be included in comparison.

However, only 112 (42%) publications followed data periods beyond the first six months of the COVID-19 pandemic (March to August 2020, inclusive). There were differences in reports of initial decrease with recovery when data was followed beyond August 2020, with only 8% recovery for shorter follow-up, increasing to 30% when follow-up was longer (latest follow-up found was through the first quarter of 2022) (**Table 4**). Examining only countries which reported any decrease (n = 212 for decrease, decrease with recovery, or mixed), reporting recovery was significantly higher when follow-up was beyond August 2020 (*P* ≤ 0.0001).

**Table 4:** Coverage data trends – pandemic periods covered*

Coverage data trends	Followed only through August 2020 (n = 152) (n (%))	Followed beyond August 2020 (n = 112) (n (%))
Decreased	70 (50%)	36 (35%)
Decreased with recovery	11 (8%)	31 (30%)
Mixed	43 (31%)	21 (20%)
Increase/stable	15 (11%)	15 (15%)
Total	139 (100%)	103 (100%)

*Only through August 2020 (i.e. first six months of pandemic assuming start of March 2020) or beyond August 2020 (i.e. beyond first six months of pandemic assuming start of March 2020).

In the 70 (27%) studies in which morbidity and/or mortality were reported, 27 (39%) noted an increase (e.g. newborn or maternal deaths) and 18 (26%) noted no change in MNCH morbidity or mortality outcomes over compared periods; nine (13%) reported mixed results (e.g. increase, decrease and/or stable) across a range of MNCH outcomes. Sixteen (22%) reports noted a decrease in morbidity and/or mortality; most were related to reductions in preterm or low birth weight infants, or reductions in childhood injuries during pandemic lockdown periods (**Table 3**).

The changing rates of low birth weight/preterm births described during COVID-19 were however, mixed overall, with seven (3%) reports noting decreased preterm/low birth weight [19-25], four (2%) reporting stable preterm/low birth weight [26-29], and two (1%) noting increased preterm/low birth weight [30,31]. Interestingly, the two reports noting increased preterm/low birth weight were both in low-income countries [30,31], while the other studies were all in high- or middle-income studies.

There were few differences in service coverage across HICs compared to LMICs. However, LMICs reported increased morbidity/mortality during COVID-19 periods (54%) compared to HIC (30%) (*P* = 0.0066) (**Table 3**).

#### Patterns or trends across countries: use of RHIS data for policy or program adjustments

Use of RHIS data for program adjustments or mitigation of indirect effects of COVID-19 was described in 58 (22%) reports, with 48 (18%) also using the RHIS data to monitor or evaluate changes following the policy and/or program responses. Mitigation efforts undertaken included increased use of telemedicine (29%), protocol or policy changes to reduce risk (e.g. shortened length of stay, (14%)), COVID-19 prevention and control measures (e.g. personal protective equipment, COVID testing pre-procedure (12%)), information campaigns and outreach (12%), change in service delivery platform or location other than telemedicine (e.g. drive-through services, 9%), and use of RHIS data for targeting response changes in EHS during the COVID-19 pandemic (9%). Eleven studies (19%) reported on the use of multiple program adjustments, primarily combinations of COVID-19 prevention and control, information campaigns, and policy or protocol changes. Of the 48 (18%) studies which reported on results of the program mitigation efforts, most reported stable (n = 14, 5%)), recovered (n = 25, 9%), or improved (n = 5, 2%)) coverage and/or morbidity/mortality; only four (1.5%) reported continued decreased service coverage despite program mitigation.

Of particular interest to this review was the use of real-time data for development of targeted response for MNCH programmes (e.g. to identify specific populations or locations which showed reduced service coverage and develop interventions informed by RHIS data). Mbithi et al. [18] used the RHIS data to identify gaps and target interventions to improve HIV/TB program coverage in Kenya; Ackerson et al. [32] reported the use of data for targeting gaps in immunization rates in the USA. Both saw improvements in coverage by using data to identify and target missed mothers or children. However, Chandir et al. [33,34] described how the Pakistan electronic immunization register (EIR) system was used to generate more default lists for catch-up of children who missed vaccinations during lockdown. The immunization program also actively engaged in implementing enhanced outreach activities and mop-up activities that were targeted at identified hotspots with highest incidence of children who missed vaccinations. Real-time EIR data was leveraged to monitor vaccinator attendance and conduct evidence-based performance management. During a period of lifted restrictions, vaccinations showed a slight two-month recovery, but immediately dropped again during the next lockdown period. Thekkur et al. [35] described how monthly real-time surveillance in 10 health facilities in Harare, Zimbabwe, was strengthened to monitor trends and target efforts to address reduced coverage of pediatric tuberculosis (TB) or human immunodeficiency virus (HIV) services during the COVID-19 pandemic, however, these targeted efforts were not successful.

## DISCUSSION

### Summary of evidence

The reports covered all six WHO regions, with most coming from Americas, Africa, and Europe. Interestingly, there was an even split across reports from HICs and LMICs, reflecting global reach and impact of COVID-19. Over 90% of the studies were from peer-reviewed sources. Only 29% included ministry or program managers as co-authors, indicating more academic approaches rather than policy-oriented program mitigation, which may limit transferability of recommendations based on the data.

Most reports compared data on MNCH services before the start of the COVID-19 pandemic with services during or after the outbreak. This is not surprising, as RHIS data usually monitor trends over time and can be valuable for comparing pre- to post/during change periods, such as seen in a public health or other emergency.

### Summary of findings by study questions

#### How was RHIS used to determine service disruption?

All WHO regions and World Bank income groups were represented, demonstrating wide use of RHIS data as a monitoring tool for health service disruptions. A WHO scoping review on interventions to maintain essential services for maternal, newborn, child health, and older people found 53 reports (during a similar review period) on programmes to mitigate impact of COVID-19 on essential services, of which 36 targeted MNCH [36], and 22% of these 36 papers used RHIS data as part of described program changes.

There did not seem to be a common approach regarding the standardization of indicators monitored across published papers in this report. In January 2021, WHO published guidance [37] suggesting RHIS indicators for monitoring effects of COVID-19 on MNCH. These could help to increase comparability of changes in RHIS data reported across settings in the current pandemic and in future ones.

There was an encouraging array of RHIS data use across both HICs and LMICs. PHC and HMIS were the most used (53%), while administrative/billing data, registries and network data were used less often. This could suggest that PHC and HMIS may be better placed for monitoring EHS disruptions, despite concerns about data quality.

Many studies introduced COVID-19 rates generally or in MNCH populations as part of the background information. Most before and after publications referred to public health measures, e.g. lockdowns, rather than COVID-19 rates. RHIS data were not linked to other data sources (e.g. laboratory or surveillance data), suggesting potential room for improvement with progressing digitalization of RHIS and interoperability of data systems.

#### Were disruptions to RHIS reporting also described (e.g. timeliness, missing data, data quality), and did that impact findings?

The WHO guidance on monitoring essential health services during COVID-19 emphasizes the need to also monitor the impact of the pandemic on program data quality [37]. Only 17% of the included reports discussed data quality (**Table 2**), which is surprising, as local routine data sources are known to have limitations due to poor data quality [38]. Quality checks of analyzed data were rarely discussed.

Only 9% of reports discussed potential data quality issues related to possible disruptions during the COVID-19 period, and very few (5%) described the quality of the RHIS data they used for their analyses. Of these, seven (3%) did not find any impact on data quality during COVID-19 periods and five (2%) reported a variety of issues. Consistent with our findings, a recent WHO report examining 19 countries noted that six reported disruptions to routine data systems during COVID-19 [39]. Impacts on the quality of RHIS data and findings of the reports on essential services are not described well in current literature, despite the WHO recommendations providing guidance on ways to assess RHIS data quality [37].

#### What are the findings on MNCH service disruptions from RHIS data analyses during COVID-19, including any patterns or trends across countries?

Most publications reported a decrease or mixed coverage results compared to pre-pandemic levels in service coverage without a recovery within the study period. A smaller proportion reported initial decreases with later recovery to pre-pandemic levels, or stable coverage across compared time periods. These data are consistent with UNICEF “pulse surveys” which reported widespread disruptions in MNCH services during the COVID-19 pandemic, including 38% reduction in maternal health services and 39% reductions in immunization services [1,2].

MNCH service coverage is a combination of both demand and supply factors, while understanding “why” coverage changes generally requires additional research, such as surveys or qualitative studies. Such studies are needed to understand changes in RHIS data and other reported changes to MNCH coverage and morbidity/mortality seen in this and other reports.

We found that studies which tracked routine data beyond August 2020 were significantly more likely to report a recovery in MNCH service coverage and conclude that services are returning to pre-pandemic levels, which is encouraging (although, as discussed above, changes possibly related to RHIS data quality were not described well in most studies). Improving routine data quality and data use to monitor coverage and morbidity/mortality will be crucial in the coming years, as mitigation programmes expand to reverse downward trends and rebuild toward reaching Sustainable Development Goals. Countries must be prepared to face additional waves of increased COVID-19 incidence with emergence of new variants and future public health emergencies.

Seventy (22%) studies reported on morbidity and/or mortality, with 39% noting an increase (e.g. increase newborn or maternal deaths, or increase mental health morbidity), 13% noting mixed, and 26% noting stable MNCH outcomes over compared periods. Sixteen (22%) countries noted decreased morbidity or mortality. These were generally either reductions in preterm/low birth weight infants or reductions in childhood injuries seen in health facilities. While a reduction in childhood injuries might be expected due to lockdowns (which kept children from school and sports), the reduction in low birth weight/preterm births is somewhat surprising. These changing rates of low birth weight/preterm births were, however, inconsistent across studies, with seven (3%) reports noting decreased preterm/low birth weight [19-24,] five (2%) reporting stable preterm/low birth weight [25-29], and two (1%) noting increased preterm/low birth weight [30,31]. Interestingly, the two reports noting increased preterm/low birth weight were both conducted in low-income countries [30,31], while the other studies were all in high- or middle-income studies. Further research to verify the impact on newborn gestational age and birthweight during the COVID-19 pandemic period is clearly needed to understand potential epidemiology and risk/protective factors for these changes during a pandemic.

We also found that LMICs reported significantly higher morbidity/mortality than HICs. This is concerning, but not unexpected, as many health systems in LMICs prior to COVID-19 faced constant challenges, so impacts due to COVID-19 on their already stretched health systems (increased lack of access, increased workloads, worsened supply and health workforce shortages) would expectedly negatively impact health outcomes beyond the direct impact of COVID-19 disease [6].

Only 58 (22%) of reports noted programmatic responses related to the impact of COVID-19 on services. The mitigation efforts undertaken by countries varied, with telemedicine being the most common. Encouragingly, among those that used RHIS data to monitor mitigation efforts, most (70%) saw improvement in response to program adjustments, suggesting substantial potential to monitor and respond to health system shocks. However, even in the reports which did not find an improvement the authors concluded that “Digital systems, like the ZM-EIR, are valuable in the response to disruptions to immunization programmes created by the pandemic” [20]. More implementation research on the use of real-time RHIS data for targeting program improvements during public health and other emergencies is needed.

### Limitations

The main limitation of this scoping review is its descriptive nature, i.e. the absence of a structured quality appraisal of included studies. Therefore, some estimates used might have come from reports which suffered substantial data limitations, which might particularly apply to gray literature and pre-publication reports. However, they made up a small percentage of included studies, while 92% of studies had at least undergone peer review.

The review was primarily conducted by one reviewer and only included full-text English language publication, possibly introducing bias. However, an indepentend consultant reviewed 20% of included and excluded reports collected by September 2021 and the study co-authors regularly reviewed all methods and results. Moreover, only five non-English language publications were found and excluded. However, gray literature government reports in non-English language could have been underrepresented, inflating the percentage from academic sources which are often published in English.

## CONCLUSIONS

RHIS are a critical component of health systems, as they provide on-going monitoring data for evidence-based decision-making and program management. This can be crucial during health and humanitarian emergencies. During the COVID-19 pandemic, RHIS have been used to collect real-time monitoring data of MNCH service coverage and health outcomes. This scoping review included 264 reports from 81 different countries using RHIS to monitor trends in service coverage and morbidity/mortality, suggesting RHIS were being used, despite potential data quality issues.

Real-time RHIS data on MNCH services could be a valuable tool for program managers and policymakers to respond to very fluid conditions of the COVID-19 pandemic and rapid changes in the virus. Results from this report and on-going work on improving RHIS with countries by WHO 36,39 and other stakeholders could inform this process for current and future public health emergencies.

Recommendations for research and practice related to using RHIS to monitor EHS for MNCH during pandemics include:

- Research on impact of pandemics on RHIS data quality and implementation of programmes to improve availability, quality, comparability, and integration of RHIS data to support real-time policy and programme responses.
- Research to understand the origins and epidemiology of coverage and morbidity/mortality changes seen during the COVID-19 pandemic (e.g. reductions in service coverage or low birth weight/preterm birth) and development of policy and programme responses to protect MNCH services and well-being during future pandemics, particularly for LMICs, as both coverage reductions and increased morbidity/mortality appeared to be higher in lower resource settings.

## Additional material


Online Supplementary Document
