# Supplementary material for: Use of routine health information systems to monitor disruptions of coverage of maternal, newborn, and child health services during COVID-19: A scoping review
Source: J Glob Health. 2023 Feb 10;13:06002. doi: 10.7189/jogh.13.06002 (PMC9910560; doi:10.7189/jogh.13.06002)
Supplement: Online Supplementary Document [file jogh-13-06002-s001.pdf]

	A	C	D	E	F	G	H	I	J	K	M	N	O	P	S	T	U	V	Y
1	**Supplemental File 1: MNCAH Services during COVID-19 using RHIS - Abstraction Sheet**																		
2																			
3	**1st Author**	**Title**	**Citation**	**MOH or Manager Authors**	**Reference Source**	**Region**	**Income Group**	**Country**	**Populations Covered**	**Topic (s)**	**RHIS Type**	**DQA**	**Data Period**	**FU beyond Sept20**	**Data Trends - MNCH Services**	**Data Trends Morb/Morality**	**Data used for program adjustments/ mitigation**	**Response Category**	**Outcome/ Impact**
4	Abdela	Essential Healthcare Services in the Face of COVID-19 Prevention: Experiences from a Referral Hospital in Ethiopia	Am. J. Trop. Med. Hyg., 103(3), 2020, pp. 1198–1200 doi:10.4269/ajtmh.20-0464	No	Peer	AFRO	LMIC	Ethiopia	MCH	EMS	Paper HMIS	None	Feb-March 22 2020 vs March 23-April 2021	No	Mixed		None		NA
5	Abdul-Mumin	Decrease in Admissions and Change in the Diagnostic Landscape in a Newborn Care Unit in Northern Ghana During the COVID-19 Pandemic	Front. Pediatr. 9:642508. doi: 10.3389/fped.2021.642508	No	Peer	AFRO	LMIC	Ghana	Newborn	Neonatal	Hosp IS	None	March to August 2019 vs 2020	No	Decrease	IncreaseMM	None		NA
6	Abebe	Trends of follow-up clinic visits and admissions three-months before and during COVID-19 pandemic at Tikur Anbessa specialized hospital, Addis Ababa, Ethiopia: an interrupted time series analysis	BMC Health Services Research (2021) 21:731 https://doi.org/10.1186/s12913-021-06730-8	No	Peer	AFRO	LMIC	Ethiopia	Children	Child Health	Hosp IS	None	follow-up visits and admissions between December 11, 2019, to June 7, 2020, with the 1st case of the COVID-19 report in Ethiopia (March 13, 2020) as a reference time.	No	Decrease		None		NA
7	Abel	Coronavirus Disease 2019 (COVID-19) andthe Incidence of Obstetric and GynecologicEmergency Department Visits in anIntegrated Health Care System	Obstet Gynecol 2021;137:581–83)DOI: 10.1097/AOG.0000000000004331	No	Peer	PAHO	HIC	USA	Maternal	EMS	EMR/HER	None	Jan-Dec 2019-2020	Yes	Decrease with recovery		None		NA
8	Ackerson	Pediatric Vaccination During the COVID-19 Pandemic	PEDIATRICS Volume 148, number 1, July 2021:e2020047092	No	Peer	PAHO	HIC	USA	Children	Immunization	EMR/HER	None	Jan-Aug 2019-2020	No	Decrease with recovery		Yes	Data for targeting	Recovery of vaccination rates
9	Ahmed	Indirect effects on maternal and child mortality from the COVID-19 pandemic: evidence from disruptions in healthcare utilization in 18 low- and middle-income countries	Lancet Preprint Available at SSRN: https://ssrn.com/abstract=3916767 or http://dx.doi.org/10.2139/ssrn.3916767	Yes	Pre-pub	AFRO, SEARO, PAHO	LMIC	Multiple	MCH	MCH	HMIS	YES DQA Overall, the completeness during the year of the pandemic for outpatient consultations is 2 percentage points greater than the pre-pandemic period and is unlikely to affect the results. As a robustness check, we present results restricted to facilities with complete reporting and show that changes in completeness during the pandemic do not drive the findings. Omissions and outliers also quantified.	pre-pandemic (January 2018 – February 2020) and the pandemic (March 2020 – June 2021) periods	No	Decrease		None		NA
10	Ahmed	The effect of COVID-19 on maternal newborn and child health (MNCH) services in Bangladesh, Nigeria and South Africa: call for a contextualised pandemic response in LMICs	International Journal for Equity in Health (2021) 20:77 https://doi.org/10.1186/s12939-021-01414-5	No	Peer	AFRO, SEARO	LMIC	Multiple	MCH	MCH	HMIS, Hosp IS	None	March-May 2019 vs 2020	No	Decrease		None		NA
11	Ainul	Trends in maternal health services in Bangladesh before, during and after CO and after COVID-19 lock VID-19 lockdowns: E downs: Evidence fr vidence from national r om national routine service data	https://knowledgecommons.popcouncil.org/departments_sbsr-rh/1299/	No	Grey	SEARO	LMIC	Bangladesh	Maternal	Mat Health	HMIS	None	2019 vs 2020	Yes	Decrease with recovery		None		NA
12	Aizawa a	Changes in childhood vaccination during the coronavirus disease 2019 pandemic in Japan	Vaccine 39 (2021) 4006–4012 https://doi.org/10.1016/j.vaccine.2021.05.050 0264-410X/	Yes	Peer	WPRO	HIC	Japan	Children	Immunization	Hosp IS	None	January and September during the years 2016–2020	Yes	Decrease with recovery		Yes	Info Campaign	Retained Childhood vaccination levels.

	A	C	D	E	F	G	H	I	J	K	M	N	O	P	S	T	U	V	Y
13	Akuaake	Cross-sectional study of paediatric case mix presenting to an emergency centre in Cape Town, South Africa, during COVID-19	BMJ Paediatrics Open 2020;4:e000801. doi:10.1136/ bmjpo-2020-000801	Yes	Peer	AFRO	LMIC	South Africa	Children	EMS	EMS IS	None	lockdown period (27 March 2020–30 April 2020) was compared with similar 5-week periods immediately before (21 February 2020–26 March 2020) and after the lockdown (1 May 2020–4 June 2020), and to similar time periods during 2018 and 2019	No	Mixed		None		NA
14	Alrabiaah	Effects of the Coronavirus disease 2019 pandemic on routine pediatric immunization coverage rates at the main University Hospital in Saudi Arabia	Saudi Med J 2020; Vol. 41 (11)	No	Peer	EMRO	HIC	Saudi Arabia	Children	Immunization	EMR/EHR	None	March-May 2017-2020	No	Decrease		None		NA
15	Alsulieman	Paediatric asthma exacerbation admissions and stringency of non-pharmaceutical interventions: Results from a developing country	IJCP 02 June 2021 https://doi-org.ez.lshtm.ac.uk/10.1111/ijcp.14423	No	Peer	EMRO	LMIC	Jordan	Children	Child Health	EMR/HER	None	Jan-Dec 2018,2019,2020	Yes		DecreaseMM	No		NA
16	Amazou	Health service utilisation during the COVID-19 pandemic in sub-Saharan Africa in 2020: a multicountry empirical assessment with a focus on maternal, newborn and child health services	BMJ Global Health 2022;7:e008069. doi:10.1136/ bmjgh-2021-008069	Yes	Peer	AFRO	LMIC	Burkina Faso, Cote d'Ivoire, Ghana, Liberia, Mali, Niger Nigeria Ethiopia, Kenya, Tanzania, Uganda and Zambia	MCH	MCH	HMIS	YES CONFIRM National completeness was over 90% in most countries during 2017–2020 and there was no major decline in monthly reporting during 2020. Nigeria is an exception with considerably lower reporting rates than the other countries.high reporting completeness and manageable number of outliers and missing values, summarised with data quality measures, as well as the consistency of findings across countries, give good confidence in the results.	March-December 2017-2020	Yes	Decrease with slight recovery		No		NA
17	Angeles	Two Years of the COVID-19 Pandemic: Estimating the Effects on Essential Health Services Utilization in Uganda and Bangladesh	https://doi.org/10.21203/rs.3.rs-1371550/v1	Yes	Pre-pub	AFRO, SEARO	LMIC	Uganda, Bangladesh	MCH	MCH	HMIS	YES Less than 0.2% of data points for each service within each country were deemed outliers. In all services in Uganda and all services in Bangladesh except for the immunization-related services, there was very little change in reporting across the time series and no change in reporting during the COVID-19 period.	Jan 2017-May 2021	Yes	Decrease with slight recovery		No		NA
18	Aranda	Disruptions in maternal health service use during the COVID-19 pandemic in 2020: experiences from 37 health facilities in low-income and middleincome countries	BMJ Global Health 2022;7:e007247. doi:10.1136/ bmjgh-2021-007247	Yes	Peer	AFRO, PAHO	LMIC	Haiti, Lesotho, Liberia, Malawi, Mexico and Sierra Leone	Maternal	Maternal Health	HMIS, EMR/HER	NO Outliers identified through Tukey's rule (larger than 1.5 times the interquartile range) were returned to the country's monitoring and evaluation officer for correction or removal.	Jan 2016-Dec2020	Yes	Decrease with recovery		Yes	Multiple	Minimal reduction with recovery
19	Arnaez	Lack of changes in preterm delivery and stillbirths during COVID-19 lockdown in a European region	European Journal of Pediatrics (2021) 180:1997–2002 https://doi.org/10.1007/s00431-021-03984-6	No	Peer	EURO	HIC	Spain	Newborn	Neonatal	Hosp IS	NO Cleaned duplicates from linked dataset	2015 til June2020	No	Stable	StableMM	No		NA
20	Arsenault	COVID-19 and resilience of healthcare systems in ten countries	Nature Medicine, https://doi.org/10.1038/s41591-022-01750-1	Yes	Peer	AFRO, PAHO, SEARO	LMIC, HIC	Haiti Ghana, Lao People's Democratic Republic, Mexico, Nepal, South Africa Thailand Chile and	MCH	MCH	HMIS	NO Cleaned missing and outliers, excluded facilities with high missing. Did not comment on whether increased missing due to COVID reporting.	Jan 2019-December 2020	Yes	Decrease with slight recovery		No		NA
21	Ashish	The perfect storm: Disruptions to institutional delivery care arising from the COVID-19 pandemic in Nepal	J Glob Health 2021;11:05010.www.jogh.org • doi: 10.7189/jogh.11.05010	Yes	Peer	SEARO	LMIC	Nepal	Maternal	Maternal Health	Hosp IS	None	March to August in 2019 with the same months in 2020).	No	Mixed		None		NA
22	Athiraman	Impact of COVID-19 on maternity and neonatal servicesThree year-on-year review data from the North East of England	Acta Paediatrica. 2022;111:1039–1041	Unk	Peer	EURO	HIC	UK	MNH	MNH	Hosp IS	None	March-June 2018, 2019, 2020	No	Mixed		Yes	Protocol Change	MM no impact

	A	C	D	E	F	G	H	I	J	K	M	N	O	P	S	T	U	V	Y
23	Ayele	Patterns of essential health services utilization and routine health information management during Covid-19 pandemic at primary health service delivery point Addis Ababa, Ethiopia.	Ethiopian Journal of Health Development ; 35(Special Issue 1):90-97, 2021. https://www.ajol.info/index.php/ejhd/issue/view/19839	No	Peer	AFRO	LMIC	Ethiopia	MCH	MCH	HMIS	YES Data accuracy and HIS performance show reductions. There was a significant decrease in Lot Quality assurance and sampling (LQAS) and Performance monitoring team (PMT) service during the COVID-19 pandemic. The reason was that most of the health workforce was partially or fully reassigned to support COVID-19 so that PMT meeting and LQAS were discontinued, and reporting was not done timely.	November 2019 to June 2020	No	Stable		Yes	Info Campaign	MCH Services were maintained but concern continues if pandemic worsens
24	Azizatunnisa',	Maintaining Polio-Free Status in Indonesia During the COVID-19 Pandemic	Glob Health Sci Pract. 2022;10(1):e2100310. https://doi.org/10.9745/GHSP-D-21-00310	Yes	Peer	SEARO	LMIC	Indonesia	Children	Immunizations	HMIS	None	2011-2020	Yes	Decrease		Yes	Multiple	NA
25	Babatunde	Impact of COVID-19 on routine immunization in Oyo State, Nigeria: Trend analysis of immunization data in the pre-and post-index case period; 2019 – 2020	https://doi.org/10.21203/rs.3.rs-308066/v1	Yes	Pre-pub	AFRO	LMIC	Nigeria	Children	Immunization	HMIS	NO Data were sorted; cleaned and relevant variables extracted	July 2019-Aug2020	No	Decrease		No		NA
26	Babu	Zambia efforts in prevention, early detection and treatment of wasting during COVID-19	https://www.ennonline.net/attachments/3760/FEX-64-Web_28Jan2021_60-63.pdf	Yes	Grey	AFRO	LMIC	Zambia	Children	Child Health	HMIS	None	Jan-Oct 2019 & 2020	Yes	Increase		Yes	Multiple	The rapid integration of COVID-19 prevention activities into the nutrition programme, through the flexibility granted by donors to redesign project activities and the prompt implementation of these measures by partners, is a key success
27	Badran	Adverse pregnancy outcomes during the COVID-19 lockdown. A descriptive study	BMC Pregnancy and Childbirth (2021) 21:761 https://doi.org/10.1186/s12884-021-04221-6	No	Peer	EMRO	LMIC	Jordan	MNH	Stillbirth	Registry	None	May 2019 to March 2020 vs April 2020 to December 2020	Yes	Mixed		No		NA
28	Bailey	The impact of COVID-19 on multi-month dispensing (MMD) policies for antiretroviral therapy (ART) and MMD uptake in 21 PEPFAR-supported countries: a multi-country analysis	Journal of the International AIDS Society 2021, 24(S6):e25794 http://onlinelibrary.wiley.com/doi/10.1002/jia2.25794/full |https://doi.org/10.1002/jia2.25794	No	Peer	AFRO, PAHO,SEARO	LMIC	Cote d'Ivoire Democratic Republic of the Congo Dominican Republic Eswatini Ethiopia	Children	HIV/TB	HMIS	None	Oct2019 Dec2020	Yes	Increase		Yes	Supply Chain, policy 3m to 6m distribution	old, virologic suppression was 71% in Q4 2019 (286,000 clients) and steadily increased to 80% in Q4 2020 (326,000 clients). Consistently increasing rates of viral suppression were maintained across nearly all
29	Banke-Thomas	A mixed-methods study of maternal health care utilisation in six referral hospitals in four sub-Saharan African countries before and during the COVID-19 pandemic	BMJ Global Health 2022;7:e008064. doi:10.1136/ bmjgh-2021-008064	Yes	Peer	AFRO	LMIC	Guinea, Nigeria, Tanzania, Uganda	Maternal	Maternal Health	HMIS	None	Mar to Feb 2019-2021	Yes	Decrease with recovery		Yes	Lockdown eased	it is important to highlight here that a perception of the pandemic being over whether self-perceived or government-induced appears to have been sufficient for change in utilisation patterns.
30	Barasa	Indirect health effects of the COVID-19 pandemic in Kenya: a mixed methods assessment	Barasa et al. BMC Health Services Research (2021) 21:740 https://doi.org/10.1186/s12913-021-06726-4	Yes	Peer	AFRO	LMIC	Kenya	MCH	MCH	HMIS	YES Assessed the data for missing values and outliers and adjusted for reporting rates. We replaced the outliner value using the median value. The quantitative analysis of changes in the level of service utilization is prone to bias from several sources including well document data quality issues of HMIS data, the likely impact of the pandemic on information systems (e.g. disruptions in reporting), and the lack of a control.	January 2019 and November 2020	Yes	Mixed		None		NA
31	Barbiellini Amidei	Pediatric emergency department visits during the COVID-19 pandemic: a large retrospective population-based study	Italian Journal of Pediatrics (2021) 47:218 https://doi.org/10.1186/s13052-021-01168-4	Yes	Peer	EURO	HIC	Italy	Children	EMS	Hosp IS	None	Jan 2019-Dec 2020	Yes	Decrease		No		NA

	A	C	D	E	F	G	H	I	J	K	M	N	O	P	S	T	U	V	Y
32	Barbieri	Antibiotic Prescription Patterns in the Paediatric Primary Care Setting before and after the COVID-19 Pandemic in Italy: An Analysis Using the AWaRe Metrics	Antibiotics 2022, 11, 457. https://doi.org/10.3390/ antibiotics11040457	No	Peer	EURO	HIC	Italy	Children	Child Health	Hosp IS	None	Feb 2019 to March 2021	Yes	Mixed		No		NA
33	Basatemur	Paediatric critical care referrals of children with diabetic ketoacidosis during the COVID-19 pandemic	Arch Dis Child April 2021 Vol 106 No 4	Yes	Peer	EURO	HIC	UK	Children	Child Health	Hosp IS	None	Jan 2018-July 2020	No		IncreaseMM	No		NA
34	Been,	Impact of COVID-19 mitigation measures on the incidence of preterm birth: a national quasi-experimental study	Lancet Public Health 2020; 5: e604–11 Published Online October 13, 2020 https://doi.org/10.1016/ S2468-2667(20)30223-1	Yes	Peer	EURO	HIC	Netherlands	Newborn	Neonatal	Hosp IS	YES Cross-validation against Perined data for selected years (2011, 2014, and 2017) showed that babies born at the lowest gestational ages and those with the lowest birthweights were consistently underrepresented in our cohort throughout the study period	Nov 2019-june 2020	No		DecreaseMM	No		NA
35	Bekele	mpact of COVID-19 Pandemic on Utilization of Facility-Based Essential Maternal and Child Health Services in North Shewa Zone, Ethiopia	https://doi.org/10.1101/2022.01.10.22268794	Yes	Pre-pub	AFRO	LMIC	Ethiopia	MCH	MCH	HMIS	None	March to August 2019 and March to August 2020	No	Decrease		No		NA
36	Bence	COVID-19 pre-procedural testing strategy and early outcomes at a large tertiary care children's hospital	Pediatric Surgery International (2021) 37:871–880 https://doi.org/10.1007/s00383-021-04878-2	No	Peer	PAHO	HIC	USA	Children	Child Health	Hosp IS	None	March 16, 2020 to October 31, 2020 compared to pre-covid average	Yes	Decrease with recovery		Yes	Epidemic Prev & Control	Recovered to pre-covid levels of procedures
37	Bents	The impact of COVID-19 non-pharmaceutical interventions on future respiratory syncytial virus transmission in South Africa	https://doi.org/10.1101/2022.03.12.22271872	No	Pre-pub	AFRO	LMIC	South Africa	Children	Child Health	Surveillance	None	January 2015-August 2021	Yes		DecreaseMM	No		NA
38	Berghella	Decreased incidence of preterm birth during coronavirus disease 2019 pandemic	American Journal of Obstetrics & Gynecology MFM Volume 4, Issue 3, May 2022, 100577 https://doi.org/10.1016/j.ajogmf. 2020.100258	No	Peer	PAHO	HIC	USA	Newborn	Neonatal	Hosp IS	None	March to July 2019 and 2020	No		DecreaseMM	No		NA
39	Bertin Ayu Wandira	Impact of the COVID-19 Pandemic on Integrated Health Service for Child (Posyandu) Management in Palu City	Macedonian Journal of Medical Sciences. 2022 Feb 20; 10(E):243-247. https://doi.org/10.3889/oamjms.2022.8149 eISSN: 1857-9655	No	Peer	SEARO	LMIC	Indonesia	MCH	MCH	HMIS	None	2018-2020	Yes	Stable		Yes	Policy	Visits maintained
40	Bhatia	Decision-to-delivery interval and neonatal outcomes for category-1 caesarean sections during the COVID-19 pandemic	Anaesthesia 2021 doi:10.1111/anae.15489	No	Peer	EURO	HIC	UK	Maternal	Mat Health	EMR/EHR & Paper MR/HR	None	1 April 2019 and 1 July 2019 compared to same period in 2020	No	Mixed	StableMM	Yes	Epidemic Prev & Control	NA
41	Bhopal	Who has been missed? Dramatic decrease in numbers of children seen for child protection assessments during the pandemic	Arch Dis Child 2021;106:e6. doi:10.1136/archdischild-2020-319783	Yes	Peer	EURO	HIC	UK	Children	Child Abuse	HMIS	None	Jan-Apr 2018-2020	No	Decrease		No		NA
42	Bogh	Nationwide study on trends in unplanned hospital attendance and deaths during the 7 weeks after the onset of the COVID-19 pandemic in Denmark	BMJ Qual Saf 2021;30:986–995. doi:10.1136/bmjqs-2020-012144	Yes	Peer	EURO	HIC	Denmark	Children	EMS	Hosp IS	None	2017 to April 2020	No	Mixed		No		NA
43	Bornstein	Early postpartum discharge during the COVID-19 pandemic	J. Perinat. Med. 2020; 48(9): 1008–1012 https://doi.org/10.1515/jpm-2020-0337	Unk	Peer	PAHO	HIC	USA	MNH	MNH	EMR	NO In addition, given the retrospective design with information based on review of medical records, not all charts had complete data available regarding pregnancy-related complications.	December 8th, 2019 and June 20th, 2020	No	Decrease		No		NA

	A	C	D	E	F	G	H	I	J	K	M	N	O	P	S	T	U	V	Y
44	Bothara	Paediatric presentations to Christchurch Hospital Emergency Department during COVID-19 lockdown	Journal of Paediatrics and Child Health 57 (2021) 877–882	Yes	Peer	WPRO	HIC	New Zealand	Children	EMS	EMR	None	Feb-Apr2018-2020	No	Decrease		No		NA
45	Bramer	Decline in Child Vaccination Coverage During the COVID-19 Pandemic — Michigan Care Improvement Registry, May 2016–May 2020	MMWR / May 22, 2020 / Vol. 69 / No. 20	Yes	Peer	PAHO	HIC	USA	Children	Immunization	EIR	None	May in 2016-2020	No	Decrease		None		NA
46	Brown	Impact of COVID-19 on Pediatric Primary Care Visits at Four Academic Institutions in the Carolinas	Int. J. Environ. Res. Public Health 2021, 18, 5734. https:// doi.org/10.3390/ijerph18115734	No	Peer	PAHO	HIC	USA	Children	EMS	EMR/EHR	None	April 2019 or April 2020	No	Decrease		None		NA
47	BT Evans	Worldwide routine immunisation coverage regressed during the first year of the COVID-19 pandemic	https://doi.org/10.1101/2021.12.03.21267195	No	Pre-pub	AFRO, SEARO, WPRO, PAHO	LMIC	India Pakistan Indonesia Philippines Mexico Uganda Peru Mozambique Argentina	Children	Immunization	HMIS	NO WUENIC	2020 Expected vs Actual	Yes	Decrease		No		NA
48	Bullinger	Pediatric emergency department visits due to child abuse and neglect following COVID-19 public health emergency declaration in the Southeastern United States	BMC Pediatrics (2021) 21:401 https://doi.org/10.1186/s12887-021-02870-2	No	Peer	PAHO	HIC	USA	Children	Child Abuse	Paper MR/HR	None	January through June 2018–2020	No	Mixed		None		NA
49	Buonsenso	Child Healthcare and Immunizations in Sub-Saharan Africa During the COVID-19 Pandemic	Front. Pediatr., 06 August 2020 | https://doi.org/10.3389/fped.2020.00517	Yes	Peer	AFRO	LMIC	Sierra Leone	Children	Immunization & Child Health	Paper HMIS	None	Jan-March 2019 vs Jan-March 2020	No	Decrease	StableMM	None		NA
50	Burger	Examining the unintended consequences of the COVID-19 pandemic on public sector health facility visits: the first 150 days	https://resep.sun.ac.za/wp-content/uploads/2021/02/16.-Examining-the-unintended-consequences-of-the-COVID-19-pandemic-on-public-sector-health-facility-visits-The-first-150-days-2.pdf	No	Grey	AFRO	LMIC	South Africa	MCH	MCH	HMIS	None	January 2018 to August 2020	No	Decrease		None		NA
51	Burt	Indirect effects of COVID-19 on maternal, neonatal, child, sexual and reproductive health services in Kampala, Uganda	BMJ Global Health 2021;6:e006102. doi:10.1136/ bmjgh-2021-006102	No	Peer	AFRO	LMIC	Uganda	MCH	MCH	Hosp IS	None	1 July 2019 and 31 December 2020,	Yes	Decrease	MixedMM	None		NA
52	Byun	The impact of the COVID-19 pandemic on outpatients of internal medicine and pediatrics	Medicine: February 25, 2022 - Volume 101 - Issue 8 - p e28884 doi: 10.1097/MD.0000000000028884	No	Peer	WPRO	HIC	South Korea	Children	Child Health	EMR	NO excluded cases without diagnosis code	January 1, 2016 and December 31, 2020.	Yes	Decrease		No		NA
53	Carré	Impact of COVID-19 lockdown on a tertiary center pediatric otolaryngology emergency department	European Journal of Pediatrics https://doi.org/10.1007/s00431-021-04236-3	No	Peer	EURO	HIC	France	Children	EMS	Hosp IS	None	March to May 2018, 2019, 2020	No	Mixed		None		NA
54	Causey	Estimating global and regional disruptions to routine childhood vaccine coverage during the COVID-19 pandemic in 2020: a modelling study	Lancet 2021; 398: 522–34 Published Online July 15, 2021 https://doi.org/10.1016/ S0140-6736(21)01337-4	No	Peer	Global	HIC, LMIC	Global	Children	Immunizations	HMIS	None	Jan-Dec 2020 comparison with expected	Yes	Decrease with recovery		No		NA

	A	C	D	E	F	G	H	I	J	K	M	N	O	P	S	T	U	V	Y
55	Chandir	Impact of COVID-19 lockdown on routine immunisation in Karachi, Pakistan	Lancetgh Vol 8 September 2020 https://doi.org/10.1016/ S2214-109X(20)30290-4	No	Peer	SEARO	LMIC	Pakistan	Children	Immunizatio n	EIR	YES Digital systems, like the ZM EIR, are valuable in the response to disruptions to immunisation programmes created by the pandemic.	baseline (Sep 23, 2019 - Mar 22, 2020), and percentage reduction during COVID-19 lockdown (Mar 23-May 9, 2020) and post COVID-19 lockdown (May 10-Jun 6, 2020)	No	Decrease		Yes	Data for targeting	Showed partila recovery, which reduced again with next lockdown
56	Chandir	Impact of COVID-19 pandemic response on uptake of routine immunizations in Sindh, Pakistan: An analysis of provincial electronic immunization registry data	Vaccine 38 (2020) 7146–7155 https://doi.org/10.1016/j.va ccine.2020.08.019 0264-410X/ 2020 Elsevier Ltd. All rights reserved	Yes	Peer	SEARO	LMIC	Pakistan	Children	Immunizatio n	EIR	NO Discussed standard issues with EIR in general and specific to province	(September 23, 2019, to March 22, 2020) was selected and compared to COVID-19 lockdown period (March 23–May 09, 2020). Some FU till July2020	No	Decrease		Yes	Data for targeting	Showed partila recovery, which reduced again with next lockdown
57	Charnaya	Effects of COVID-19 pandemic on pediatric kidney transplant in the United States	Pediatric Nephrology https://doi.org/10.1007/s00 467-020-04764-4	Yes	Peer	PAHO	HIC	USA	Children	Specialty	Registry	None	Feb-Jun 2019 vs 2020	No	Decrease with recovery		None		NA
58	Chaziya	COVID-19 in Malawi: lessons in pandemic preparedness from a tertiary children's hospital	Arch Dis Child 2021;106:238–240. doi:10.1136/archdischild-2020-319980	No	Peer	AFRO	LMIC	Malawi	Children	EMS	Hosp IS	None	Jan-Aug 2019 vs 2020	No	Decrease	IncreaseMM	None		NA
59	Chelo	Impact and projections of the COVID-19 epidemic on attendance and routine vaccinations at a pediatric referral hospital in Cameroon	Archives de Pe´diatrie 28 (2021) 441–450 https://doi.org/10.1016/j.ar cped.2021.05.006 0929-693X/	Yes	Peer	AFRO	LMIC	Cameroon	MCH	MCH	Hosp IS	None	January 1, 2016, to May 31, 2020.	No	Decrease		None		NA
60	Chimamise	Impact of Covid-19 pandemic on obstetric fistula repair program in Zimbabwe	PLoS ONE 16(4): e0249398. https://doi.org/ 10.1371/journal.pone.02493 98	No	Peer	AFRO	LMIC	Zimbabwe	Maternal	Mat Health	Registry	None	2019 vs 2020	Yes	Decrease		None		NA
61	Chisini	COVID-19 pandemic impact on paediatric dentistry treatments in the Brazilian Public Health System	Int J Paediatr Dent. 2021;31:31–34 DOI: 10.1111/ipd.12741	No	Peer	PAHO	LMIC	Brazil	Children	Specialty	Network Database	None	January 2019 to May 2020	No	Decrease		None		NA
62	Chiu	Changes in pediatric seizure-related emergency department attendances during COVID-19 e A territory-wide observational study	Journal of the Formosan Medical Association 120 (2021) 1647e1651	No	Peer	WPRO	LMIC	China	Children	EMS	Hosp IS	None	January 23 and April 22 in 2015e2020,	No	Mixed		No		NA
63	Chong	Impact of COVID-19 on pediatric emergencies and hospitalizations in Singapore	BMC Pediatrics (2020) 20:562 https://doi.org/10.1186/s12 887-020-02469-z	No	Peer	SEARO	HIC	Singapore	Children	EMS	EMR/EHR	None	Jan-Aug 2020	No	Mixed		None		NA
64	Connolly	Childhood immunization during the COVID-19 pandemic: experiences in Haiti, Lesotho, Liberia and Malawi	Bull World Health Organ 2022;100:115–126C| doi: http://dx.doi.org/10.2471/B LT.21.286774	Yes	Peer	AFRO, PAHO	LMIC	Haiti, Lesotho, Liberia, Malawi	Children	Immunizatio n	HMIS	None	March2020-Aug 2021	Yes	Decrease with recovery		Yes	Logistic Support and Incentives	Recovery of vaccination rates
65	Coulibaly-Zerbo	Maintaining Essential Nutrition Services to Underfive Children in Yemen: A Programmatic Adaptation Amidst the COVID-19 Pandemic	Children 2021, 8, 350. https:// doi.org/10.3390/children80 50350	No	Peer	EMRO	LMIC	Yemen	Children	Child Health	HMIS	None	2018-2020	Yes	Increase	IncreaseMM	No		NA
66	Courtney	Decreases in Young Children Who Received Blood Lead Level Testing During COVID-19 — 34 Jurisdictions, January–May 2020	MMWR / February 5, 2021 / Vol. 70 / No. 5	No	Peer	PAHO	HIC	USA	Children	Specialty	Surveillance	None	January–May 2019 and January–May 2020.	No	Decrease		No		NA

	A	C	D	E	F	G	H	I	J	K	M	N	O	P	S	T	U	V	Y
67	Crockett	Rapid Conversion from Clinic to Telehealth Behavioral Services During the COVID-19 Pandemic	https://doi.org/10.1007/s40617-020-00499-8 / Published online: 15 October 2020 Behavior Analysis in Practice (2020) 13:725–735	Yes	Peer	PAHO	HIC	USA	Children	Mental Health	HMIS	None	Jan-June2020	No	Decrease with Recovery		Yes	Telemedicine	Recovery of total contacts
68	Dandena	Impact of COVID-19 and mitigation plans on essential health services: institutional experience of a hospital in Ethiopia	BMC Health Services Research (2021) 21:1105 https://doi.org/10.1186/s12913-021-07106-8	Yes	Peer	AFRO	LMIC	Ethiopia	MCH	MCH	Paper HMIS	None	May-Oct 2019 & 2020	Yes	Decrease with recovery		Yes	Multiple	Less impact on Maternity and Emergency services
69	das Neves Martins Pires	Covid-19 pandemic impact on maternal and child health services access in Nampula, Mozambique: a mixed methods research	BMC Health Services Research (2021) 21:860 https://doi.org/10.1186/s12913-021-06878-3	No	Peer	AFRO	LMIC	Mozambique	MCH	MCH	HMIS	None	March-May 2019 vs 2020	No	Mixed		None		NA
70	Davico	Where have the children with epilepsy gone? An observational study of seizure-related accesses to emergency department at the time of COVID-19	Seizure: European Journal of Epilepsy 83 (2020) 38–40 https://doi.org/10.1016/j.seizure.2020.09.025	No	Peer	EURO	HIC	Italy	Children	EMS	Paper MR/HR	None	January 6, 2020 and April 21, 2020, were examined and compared with the corresponding periods of 2019.	No	Decrease		None		NA
71	de Carvalho-Sauer	Impact of COVID-19 pandemic on time series of maternal mortality ratio in Bahia, Brazil: analysis of period 2011–2020	BMC Pregnancy Childbirth (2021) 21:423 https://doi.org/10.1186/s12884-021-03899-y	Yes	Peer	PAHO	LMIC	Brazil	Maternal	Mat Health	HMIS	YES Discuss secondary data use and possible delayed reporting	2013-2019 vs 2020	Yes		IncreaseMM	None		NA
72	De Curtis	Increase of stillbirth and decrease of late preterm infants during the COVID-19 pandemic lockdown	Arch Dis Child Fetal Neonatal Ed 2021;106:F456. doi:10.1136/fetalneonatal-2020-320682	Yes	Peer	EURO	HIC	Italy	Newborn	Neonatal	Hosp IS	None	(March, April, May 2020 & 2019	No		MixedMM	No		NA
73	de Oliviera	Repercussions of the COVID-19 pandemic on preventive health services in Brazil	Preventive Medicine 155 (2022) 106914	No	Peer	PAHO	LMIC	Brazil	Children	Immunizations	HMIS	None	March-Dec 2017-2020	Yes	Decrease		No		NA
74	Delaroche	Pediatric Emergency Department Visits at US Children's Hospitals During the COVID-19 Pandemic	PEDIATRICS Volume 147, number 4, April 2021:e2020039628 ARTICLE Downloaded from http://publications.aap.org/pediatrics/article-pdf/147/4/e2020039628/1182017/peds_2020039628.pdf	No	Peer	PAHO	HIC	USA	Children	EMS	Hosp IS	None	March 15 to August 31, 2017–2020	No	Decrease		No		NA
75	DeSilva	Association of the COVID-19 Pandemic With Routine Childhood Vaccination Rates and Proportion Up to Date With Vaccinations Across 8 US Health Systems in the Vaccine Safety Datalink	JAMA Pediatr. 2022;176(1):68-77. doi:10.1001/jamapediatrics.2021.4251	Yes	Peer	PAHO	HIC	USA	Children	Immuniztion	EIR	None	Jan-Oct 2019-2020	Yes	Decrease		No		NA
76	Desta	Impacts of COVID-19 on essential health services in Tigray, Northern Ethiopia: A prepost study	PLoS ONE 16(8): e0256330. https://doi.org/10.1371/journal.pone.0256330	Yes	Peer	AFRO	LMIC	Ethiopia	MCH	MCH	HMIS	NO Discussed Reporting quality generally	second quarter of 2020 (Post COVID19) compared to similar quarter in 2019 (Pre COVID-19) (3 months period). T	No	Mixed		None		NA
77	Dong	Routine childhood vaccination rates in an academic family health team before and during the first wave of the COVID-19 pandemic: a pre–post analysis of a retrospective chart review	CMAJ Open 2022 January 25. DOI:10.9778/cmajo.20210084	No	Peer	PAHO	HIC	Canada	Children	Immunization	EMR	None	Jan. 1, 2018, and Aug. 31, 2020	No	Decrease		No		NA
78	Dopfer	COVID-19 related reduction in pediatric emergency healthcare utilization – a concerning trend	BMC Pediatrics (2020) 20:427 https://doi.org/10.1186/s12887-020-02303-6	No	Peer	EURO	HIC	Germany	Children	EMS	EMR/EHR	None	Jan-Apr 2019 vs 2020	No	Decrease	StableMM	None		NA
79	Dorward	The impact of the COVID-19 lockdown on HIV care in 65 South African primary care clinics: an interrupted time series analysis	Lancet HIV 2021; 8: e158–65 Published Online February 4, 2021 https://doi.org/10.1016/S2352-3018(20)30359-3	Yes	Peer	AFRO	LMIC	South Africa	Children	HIV/TB	HMIS	None	Jan 1, 2018, to July 31, 2020	No	Decrease with recovery		None		NA

	A	C	D	E	F	G	H	I	J	K	M	N	O	P	S	T	U	V	Y
80	Doubova	Overcoming disruptions in essential health services during the COVID-19 pandemic in Mexico	BMJ Global Health 2022;7:e008099. doi:10.1136/ bmjgh-2021-008099	Yes	Peer	PAHO	LMIC	Mexico	MCH	MCH	HMIS	None	January 2019 to August 2021.	Yes	Decrease with slight recovery		Yes	Multiple	reovery but not to prepandemic
81	Doubova	Disruption in essential health services in Mexico during COVID-19: an interrupted time series analysis of health information system data	BMJ Global Health 2021;6:e006204. doi:10.1136/ bmjgh-2021-006204	No	Peer	PAHO	LMIC	Mexico	MCH	MCH	HMIS	YES Discussed routine DQA in methods and noted in discussion "The pandemic may have affected reporting quality of the data. However, the health information division did not report changes in data completeness and quality."	for January 2019 to December 2020	Yes	Decrease		None		NA
82	Du	Association between the COVID-19 pandemic and the risk for adverse pregnancy outcomes: a cohort study	BMJ Open 2021;11:e047900. doi:10.1136/ bmjopen-2020-047900	Yes	Peer	WPRO	LMIC	China	MNH	MNH	Hosp IS	None	Jan-Dec 2019 vs Jan-Jul2020	No		IncreaseMM	None		NA
83	Dvir,	Comparison of Use of the Massachusetts Child Psychiatry Access Program and Patient Characteristics Before vs During the COVID-19 Pandemic	JAMA Network Open. 2022;5(2):e2146618. doi:10.1001/jamanetworkopen.2021.46618 (Repr	Yes	Peer	PAHO	HIC	USA	Children	Mental Health	HMIS	None	2019 through 2021	Yes	Decrease with recovery		No		NA
84	Elalouf	Pediatric Dental Emergency Visits and Treatment during Lockdown in the COVID-19 Pandemic: A Retrospective Study	Int. J. Environ. Res. Public Health 2022, 19, 3774. https://doi.org/10.3390/ ijerph19073774	No	Peer	EMRO	HIC	Israel	Children	EMS	Hosp IS	None	(2019 and 2020) between 19 March and 30 April and after the lockdown period from 1 May to 12 June 2020.	No	Mixed		Yes	PPE, Telemedicine, Screening	adjusted rapidly to pandemic situation
85	Emmanuel	ndirect effects of COVID-19 pandemic on reproductive, maternal, newborn and child health services in Pakistan	EMHJ – Vol. 28 No. 4 – 2022	Yes	Peer	SEARO	LMIC	Pakistan	MCH	MCH	Hosp IS	None	May-Dec 2019-2020	Yes	Decrease with Recovery		No		NA
86	Enbiale,	Effect of the COVID-19 Pandemic Preparation and Response on Essential Health Services in Primary and Tertiary Healthcare Settings of Amhara Region, Ethiopia	Am. J. Trop. Med. Hyg., 105(5), 2021, pp. 1240–1246 doi:10.4269/ajtmh.21-0354	Yes	Peer	AFRO	LMIC	Ethiopia	MCH	MCH	Paper MR/HR	None	July 7, 2019 to July 6, 2020	No	Decrease with recovery		Yes	Info Campaign & Policy	We think that the reasons for the sustained and recovery of the essential health service are two factors. Firstly, the swift response by the MOH and regional health bureaus and secondly, the slow progression of the pandemic which never overwhelmed
87	Erdmann	Impact of the COVID-19 pandemic on incidence, time of diagnosis and delivery of healthcare among paediatric oncology patients in Germany in 2020: Evidence from the German Childhood Cancer Registry and a qualitative survey	The Lancet Regional Health - Europe (2021), https://doi.org/10.1016/j.lanepe.2021.100188	No	Peer	Euro	HIC	Germany	Children	Specialty	Registry	None	2015-2019 vs 2020	Yes	Mixed		None		NA
88	Ferrero	Impact of the COVID-19 pandemic in the paediatric emergency department attendances in Argentina	Arch Dis Child 2021;106:e5.	Yes	Peer	PAHO	LMIC	Argentina	Children	EMS	Hosp IS	None	January to May) of 2019 and 2020	No	Decrease		No		NA
89	Fidancı	The impact of the COVID-19 pandemic on paediatric emergency service	Int J Clin Pract. 2021;75:e14398. wileyonlinelibrary.com/journal/ijcp | 1 of 7 https://doi.org/10.1111/ijcp.14398	No	Peer	EURO	HIC	Turkey	Children	EMS	EMR	None	2019 and 2020 from the months of April to October	Yes	Decrease	IncreaseMM	No		NA
90	Fitrianingrum	Health Impact Assessment of Covid-19 Towards Maternal Health Care in West Jakarta	Journal of Maternal and Child Health (2021), 06(02): 229-237; http://thejmch.com/index.php?journal=thejmch&page=article&op=view&path%5B%5D=557&path%5B%5D=pdf	No	Peer	SEARO	LMIC	Indonesia	Maternal	Mat Health	HMIS	None	January 2018-May2020	No	Decrease with recovery		None		NA

	A	C	D	E	F	G	H	I	J	K	M	N	O	P	S	T	U	V	Y
91	Franzolin	Pediatric eye emergency department activity during the first wave of Covid-19 pandemic	Italian Journal of Pediatrics (2021) 47:217 https://doi.org/10.1186/s13052-021-01167-5	No	Peer	EURO	HIC	Italy	Children	EMS	EMR	None	March to May 2017-2020	No	Decrease		No		NA
92	Garstang	Effect of COVID-19 lockdown on child protection medical assessments: a retrospective observational study in Birmingham, UK	BMJ Open 2020;10:e042867. doi:10.1136/bmjopen-2020-042867	Yes	Peer	EURO	HIC	UK	Children	Child Abuse	Paper MR/HR	None	18-week period from late February to late June during the years 2018–2020.	No	Decrease		None		NA
93	Gebreegziabher	Assessment of maternal and child health care services performance in the context of COVID-19 pandemic in Addis Ababa, Ethiopia: evidence from routine service data	Reproductive Health (2022) 19:42 https://doi.org/10.1186/s12978-022-01353-6	No	Peer	AFRO	LMIC	Ethiopia	MCH	MCH	HMIS	NO Routine only	July 2019 to March 2021	Yes	Decrease with recovery		No		NA
94	Gerall	Delayed presentation and sub-optimal outcomes of pediatric patients with acute appendicitis during the COVID-19 pandemic	Journal of Pediatric Surgery 56 (2021) 905–910	No	Peer	PAHO	HIC	USA	Children	EMS	EMR	None	March to May 2019-2020	No		IncreaseMM	No		NA
95	Gill	Reasons for Admissions to US Children's Hospitals During the COVID-19 Pandemic	JAMA April 27, 2021 Volume 325, Number 16 (	No	Peer	PAHO	HIC	USA	Children	EMS	Hosp IS	None	March-Aug 2017-2020	No	Decrease		No		NA
96	Glazier	Shifts in office and virtual primary care during the early COVID-19 pandemic in Ontario, Canada	CMAJ 2021 February 8;193:E200-10. doi: 10.1503/cmaj.202303	No	Peer	PAHO	HIC	Canada	Children	Child Health	Admin/Billing	None	Jan. 1 to July 28, 2020, with the same period in 2019	No	Mixed		None		NA
97	Golandaj	Pediatric TB detection in the era of COVID-19	Indian Journal of Tuberculosis, https://doi.org/10.1016/j.ijtb.2021.04.015	Yes	Peer	SEARO	LMIC	India	Children	HIV/TB	HMIS	None	January to September for the year 2020 were compared to 2019	Yes	Decrease		None		NA
98	Gomez et.al.	A population-based analysis of the impact of the COVID-19 pandemic on common abdominal and gynecological emergency department visits	CMAJ 2021 May 25;193:E753-60. doi: 10.1503/cmaj.202821	No	Peer	PAHO	HIC	Canada	Maternal	EMS	EMS IS	None	Jan. 1, 2019–July 1, 2019, and Jan. 1, 2020–June 30, 2020.	No	Decrease		None		NA
99	Güemes	Severity in pediatric type 1 diabetes mellitus debut during the COVID-19 pandemic	J Pediatr Endocrinol Metab 2020; 33(12): 1601–1603	Yes	Peer	EURO	HIC	Spain	Children	Specialty	Hosp IS	None	March 21st and May 6th 2020, 2018, 2019	No		IncreaseMM	No		NA
100	Gurol-Urganci	Obstetric interventions and pregnancy outcomes during the COVID-19 pandemic in England: Anationwide cohort study	PLoS Med19(1): e1003884. https://doi.org/10.1371/journal.pmed.1003884	Yes	Peer	EURO	HIC	UK	MNH	MNH	Hosp IS	None	23 March 2020 to 22 February 2021) and the pre-pandemic period (the corresponding calendar period 1year earlier: 23 March 2019 to 22 February 2020	Yes	Mixed	StableMM	No		NA
101	HA Taner	How did the COVID-19 pandemic affect child and adolescent psychiatry outpatient clinic admissions? A single-center, retrospective study	Clinical Child Psychology and PsychiatryVolume 27, Issue 3, July 2022, Pages 824-835 https://doi.org/10.1177/13591045221095657	No	Peer	EURO	HIC	Turkey	Children	Mental Health	EMR	None	March 2019 to March 2020 (pre-pandemic) and from March 2020 to March 2021	Yes	Mixed		No		NA
102	Haddadin	Changes in Pediatric Emergency Department Visits During the COVID-19 Pandemic	HOSPITAL PEDIATRICS Volume 11, Issue 4, April 2021 DOI:https://doi.org/10.1542/hpeds.2020-005074	No	Peer	PAHO	HIC	USA	Children	EMS	Hosp IS	None	2018, 2019, 2020	Yes	Mixed		None		NA
103	Hadley et.al.	EMS activations by pregnant patients in Maryland (USA) during the COVID-19 pandemic.	Prehosp Disaster Med. 2021;00(00):1–6.	No	Peer	PAHO	HIC	USA	Maternal	EMS	EMS IS	None	pandemic period (March 10, 2020 through July 20, 2020) to the pre-pandemic period during the same time frame in the prior year (March 10, 2019 through July 20, 2019)	No	Decrease	StableMM	None		NA

	A	C	D	E	F	G	H	I	J	K	M	N	O	P	S	T	U	V	Y
104	Handley	Changes in Preterm Birth Phenotypes and Stillbirth at 2 Philadelphia Hospitals During the SARS-CoV-2 Pandemic, March-June 2020	JAMA January 5, 2021 Volume 325, Number 1 87	No	Peer	PAHO	HIC	USA	Newborn	Neonatal	Surveillance	None	March through June of 2018, 2019, and 2020	No		StableMM	No		NA
105	Hans	Impact of Pandemic on Women Health Indicators in a Tertiary Health Centre of Bihar	J Evolution Med Dent Sci 2021;10(22):1639-1644, DOI: 10.14260/jemds/2021/341	Yes	Peer	SEARO	LMIC	India	MNH	MNH	Paper MR/HR	None	April 2019 to September 2019, total of 20,961 were in "pre Covid-19" control group, while patients seen from April 2020 to September 2020,	Yes	Decrease	IncreaseMM	No		NA
106	Haqqi	COVID-19 in Pakistan: Impact on global polio eradication initiative	J Med Virol. 2021;93:141–143.	Yes	Peer	SEARO	LMIC	Pakistan	Children	Immunizations	HMIS	None	2014-June2020	No		IncreaseMM	No		NA
107	Harris	Effects of the Coronavirus Disease 2019 Pandemic on Human Immunodeficiency Virus Services: Findings from 11 Sub-Saharan African Countries	CID 2022 https://doi.org/10.1093/cid/ciab951	Yes	Peer	AFRO	LMIC	Angola, DRC, Eswatini, Ethiopia, Kenya, South Sudan, Burundi, Cameroon, Cote d'Ivoire, Mozambique, Zambia	Maternal	HIV/TB	HMIS	None	Q1 (October 2019–December 2019) and Q2 (January 2020–March 2020) data reflecting HIV service delivery provision before the implementation of any COVID-19–related restrictions. Q3 (April 2020–June 2020) data reflected HIV service delivery during the time period of restrictions, and Q4 (July 2020–September 2020)	Yes	Decrease with recovery		No		NA
108	Hartford	Pediatric Emergency Department Responses to COVID-19: Transitioning From Surge Preparation to Regional Support	Disaster Medicine and Public Health PreparednessVOL. 15/NO. 1DOI: 10.1017/dmp.2020.197	Yes	Peer	PAHO	HIC	USA	Children	EMS	Hosp IS	None	Dec2019-Apr2020	No	Decrease		No		NA
109	Hategeka	Impact of the COVID-19 pandemic and response on the utilisation of health services in public facilities during the first wave in Kinshasa, the Democratic Republic of the Congo	BMJ Global Health 2021;6:e005955. doi:10.1136/ bmjgh-2021-005955	No	Peer	AFRO	LMIC	DRC	MCH	MCH	HMIS	YES Discussed regular DQA activities, note substantial missing data but not related to COVID	pre-COVID-19 period (January 2018 to February 2020) and the COVID-19 period (March to December 2020).	Yes	Mixed		None		NA
110	Hedermann	Danish premature birth rates during the COVID-19 lockdown	Arch Dis Child Fetal Neonatal Ed 2021;106:F93–F95. doi:10.1136/archdischild-2020-319990	No	Peer	EURO	HIC	Denmark	Newborn	Neonatal	Surveillance	None	(12 March–14 April 2020) and in the previous 5 years (2015–2019)	No		DecreaseMM	No		NA
111	Hedstrom	Impact of the early COVID-19 pandemic on outcomes in arural Ugandan neonatal unit: Aretrospective cohort study	PLoS ONE16(12): e0260006. https://doi.org/10.1371/journal. pone.0260006	Yes	Peer	AFRO	LMIC	Uganda	Newborn	Neonatal	Hosp IS	None	(September 2019 toMarch 2020) and during the early COVID-19 period (April–September 2020)	Yes		IncreaseMM	No		NA
112	Hekimo▯glu	Effects of COVID-19 pandemic period on neonatal mortality and morbidity	Pediatrics and Neonatology 63 (2022) 78e83	Yes	Peer	EURO	HIC	Turkey	Newborn	Neonatal	Paper MR/HR	None	1 March and 30 May 2020 & 2019	No	Decrease	MixedMM	No		NA
113	Howarth	Trends in healthcare utilisation during COVID-19: a longitudinal study from the UK	BMJ Open 2021;11:e048151. doi:10.1136/ bmjopen-2020-048151	Yes	Peer	EURO	HIC	Turkey	Maternal	Maternal Health	Admin/Billing	None	January 2018 to August 2020	No	Stable		No		NA
114	Hui	Effect of COVID-19 on delivery plans and postnatal depression scores of pregnant women	Hong Kong Med J 2020;26:113–7 https://doi.org/10.12809/hkmj208774	No	Peer	WPRO	LMIC	China (Hong Kong)	Maternal	Mental Health	Hosp IS	None	pre-alert period (1 Jan 2019 to 4 Jan 2020) and the post-alert period (5 Jan 2020 to 30 Apr 2020).	No	Mixed		None		NA
115	Hull	The impact of the COVID-19 pandemic on routine vaccinations in Victoria	MJA 215 (2) • 19 July 2021	Yes	Peer	WPRO	HIC	Australia	Children	Immunization	EIR	None	Jan 2019-Oct2020	Yes	Stable		Yes	Change Service Platform	HPV dose 1 vaccination of adolescents declined noticeably during the first but not the second epidemic wave
116	Hurst	Reduced pediatric urgent asthma utilization and exacerbations during the COVID-19 pandemic	Pediatric Pulmonology. 2021;56:3166–3173.	No	Peer	PAHO	HIC	USA	Children	Specialty	EMR	None	March 1, 2019 through February 28, 2021	Yes		DecreaseMM	No		NA

	A	C	D	E	F	G	H	I	J	K	M	N	O	P	S	T	U	V	Y
117	J Grudziz-Sekowska	Healthcare Utilization and Adherence to Treatment Recommendations among Children with Type 1 Diabetes in Poland during the COVID-19 Pandemic	J. Environ. Res. Public Health 2022, 19, 4798. https://doi.org/10.3390/ijerph19084798	No	Peer	EURO	HIC	Poland	Children	Specialty	Admin/Billing	None	2016–2020	Yes	Decrease	IncreaseMM	No		NA
118	Jenner	Kids are back in town: the return of high demand for paediatric emergency care	Arch Dis Child 2021;0:1. doi:10.1136/archdischild-2021-322298	Yes	Peer	EURO	HIC	UK	Children	EMS	Hosp IS	None	April 2020–May 2021 vs 2019-2020	Yes	Decrease with recovery		No		NA
119	Jensen	Child health services during a COVID-19 outbreak in KwaZulu-Natal Province, South Africa	S Afr Med J 2021;111(2):114-119. https://doi.org/10.7196/SAMJ.2021.v111i2.15243	Yes	Peer	AFRO	LMIC	South Africa	MCH	MCH	Hosp IS	None	January 2018 - June 2020	No	Mixed	IncreaseMM	Yes	Info Campaign	Immun rates increased
120	Jensen	The feasibility and ongoing use of electronic decision support to strengthen the implementation of IMCI in KwaZulu-Natal, South Africa	BMC Pediatrics (2022) 22:80 https://doi.org/10.1186/s12887-022-03147-y	Yes	Peer	AFRO	LMIC	South Africa	Children	Child Health	EMR	None	December 2019 – January 2021	Yes	Decrease		Yes	Telemedicine	NA
121	Jensen	Child health services during a COVID-19 outbreak in KwaZulu-Natal Province, South Africa	S Afr Med J 2021;111(2):114-119. https://doi.org/10.7196/SAMJ.2021.v111i2.15243	Yes	Peer	AFRO	LMIC	South Africa	Children	Child Health	HMIS	YES Data Outliers were identified, and numbers were verified and corrected with district information officers. potential inaccuracies in the DHIS data set. The routine DHIS data collection and capturing process involves multiple manual and electronic steps, leaving room for human error. Several levels of data verification are meant to mitigate the risk of inaccuracy,[26] but such routines may have been conducted less regularly during the COVID-19 outbreak.	January 2018 - June 2020	No	Decrease	IncreaseMM	None		NA
122	Ji	Impact of the COVID-19 pandemic on routine immunization coverage in children under 2 years old in Ontario, Canada: A retrospective cohort study	https://doi.org/10.1101/2021.10.28.21265578	Yes	Pre-pub	PAHO	HIC	Canada	Children	Immunizations	EMR	None	January 2019-March 2020 (T1); March-July 2020 (T2); and August-December 2020 (T3).	Yes	Decrease with slight recovery		No		NA
123	Jiang	Equal Access to Telemedicine during COVID-19 Pandemic: A Pediatric Otolaryngology Perspective	Laryngoscope, 00:1–5, 2020 DOI: 10.1002/lary.29164	No	Peer	PAHO	HIC	USA	Children	Specialty	EMR/EHR & Census	None	6-week period between March and May 2020 compared to same period in 2019	No	Stable		Yes	Telemedicine	Stable outcomes; Were able to completely change to telemedicine during lockdown;
124	Jiarong Pan	Impact of the COVID-19 pandemic on infectious disease hospitalizations of neonates at a tertiary academic hospital: a cross-sectional study	BMC Infectious Diseases (2022) 22:206 https://doi.org/10.1186/s12879-022-07211-x	No	Peer	WPRO	LMIC	China	Newborn	Neonatal	Hosp IS	None	January 2015 to December 2020	Yes	Decrease		No		NA
125	Jiee	Polio Supplementary Immunization Activities During COVID-19 Pandemic: Experience from Penampang District, Sabah, Malaysia	Journal of Primary Care & Community Health Volume 12: 1–9 DOI: 10.1177/21501327211029800	Yes	Peer	SEARO	LMIC	Malasia	Children	Immunization	HMIS	None	Jan 2020 to Jan 2021	Yes	Stable		Yes	Change Service Platform	90% coverage of intended SIA
126	Jones	Population birth outcomes in 2020 and experiences of expectant mothers during the COVID-19 pandemic: a 'Born in Wales' mixed methods study using routine data	medRxiv preprint doi: https://doi.org/10.1101/2021.08.23.21262209; t	Yes	Pre-pub	EURO	HIC	UK	MCH	MCH	EMR/EHR	None	infants born in 2020 compared to 2016- 2019	Yes	Mixed		None		NA
127	K Schweiberger	Trends in Pediatric Primary Care Visits During the Coronavirus Disease of 2019 PandemicT	academic ped Volume 21, Number 8 November–December 2021	No	Peer	PAHO	HIC	USA	Chidlren	Child Health	Admin/Billing	YES Due to potential lag in claims data, we tested the completeness of claims by calculating weekly rates of childbirths per 1000 female enrollees between 15 and 44 years old and confirmed that the weekly rate of childbirth in the data remained stable from January 1 through October 6, 2020.14,15 To evaluate whether the substantial rise in unemployment during the pandemic was associated with increased insurance drop-out rates during 2020 compared to prior years, we examined insurance drop-out rates across the 3 years and found the drop-out rate was similar for each year.T	January–October 2018 and 2019, 2020	Yes	Decrease with Recovery	DecreaseMM	Yes	Multiple	clinics worked to institute a range of strategies to support physical distancing in the office (eg, restricting the number of appointments, utilizing telemedicine visits, limiting types of conditions seen, maintaining adequate supply of personal protective equipment)17,34–36 which may contribute to the increased visit rates during the later pandemic period studied.T

	A	C	D	E	F	G	H	I	J	K	M	N	O	P	S	T	U	V	Y
128	Kasonia	The impact of the COVID-19 pandemic on the provision & utilisation of primary health care 1 services in Goma, Democratic Republic of the Congo, Kambia district, Sierra Leone & Masaka 2 district, Uganda	https://www.medrxiv.org/content/10.1101/2022.04.28.22274416v1	Yes	Pre-pub	AFRO	LMIC	DRC, Sierra Leone, Uganda	MCH	MCH	Paper HMIS	None	1st January 2018 to the 27th December 2020.	Yes	Mixed		Yes	Multiple	reovery for most variables,
129	Kassie	Impact of Coronavirus Diseases-2019 (COVID-19) on Utilization and Outcome of Reproductive, Maternal, and Newborn Health Services at Governmental Health Facilities in South West Ethiopia, 2020: Comparative Cross-Sectional Study	International Journal of Women's Health 2021:13 479–488	No	Peer	AFRO	LMIC	Ethiopia	MNH	MNH	Paper MR/HR	None	March–June 2019 and March–June 2020	No	Mixed	IncreaseMM	None		NA
130	Katz	One year into COVID-19: What have we learned about child maltreatment reports and child protective service responses?	Child Abuse & Neglect https://doi.org/10.1016/j.chiabu.2021.105473	Yes	Peer	AFRO, WPRO, PAHO, EURO, EMRO	HIC, LMIC	Australia Brazil USA Colombia Germany Israel Japan Canada South Africa England	Children	Mental Health	HMIS	None	March-Dec 2020	Yes		IncreaseMM	No		NA
131	Kemme	Decreased access to pediatric liver transplantation during the COVID-19 pandemic	Pediatric Transplantation. 2022;26:e14162. https://doi.org/10.1111/petr.14162	No	Peer	PAHO	HIC	USA	Chidlren	Specialty	Surveillance	None	pre-COVID-19 (March-November 2016–2019), early COVID-19 (March-May 2020), and late COVID-19 (June-November 2020).	Yes	Decrease with recovery		No		NA
132	Kenney	Demand for regional level III neonatal services is not reduced during national COVID lockdowns	Early Human Development Volume 163, December 2021, 105491	Yes	Peer	EURO	HIC	UK	Newborn	Neonatal	EMR	None	March-June 2016-2020; Jan-March 2017-2021	Yes	Stable		No		NA
133	Khan	Impact of COVID-19 Pandemic on Childhood Immunization in a Tertiary Health-Care Center	Indian J Community Med. 2021 Jul-Sep; 46(3): 520–523. Published online 2021 Oct 13. doi: 10.4103/ijcm.IJCM_847_20	Yes	Peer	SEARO	LMIC	India	Children	Immunization	HMIS	None	Jan-July 2019-2020	No	Decrease		No		NA
134	Khazaeipour	Indirect efects of COVID-19 in referring women to gynecologic oncology, perinatology and gynecology clinics in Iran	Archives of Gynecology and Obstetrics (2021) 304:679–686 https://doi.org/10.1007/s00404-021-06097-5	No	Peer	EMRO	LMIC	Iran	Maternal	Mat Health	Hosp IS	None	February 20 to May 20 in 2020 vs same in 2019	No	Decrease		None		NA
135	Khazanchi,	Trends in Pediatric Viral Symptoms, Influenza Testing, and SARS-CoV-2 Testing From a Statewide Electronic Health Record Consortium, January 2017 to July 2021	Academic Ped Volume 21, Number 8 November–December 2021	Yes	Peer	PAHO	HIC	USA	Children	Child Health	EMR	None	January 1, 2017 through July 30, 2021	Yes	Decrease	DecreaseMM	No		NA
136	Kiely	COVID-19 pandemic impact on childhood vaccination coverage in Quebec, Canada	HUMAN VACCINES & IMMUNOTHERAPEUTICS2022, VOL. 18, NO. 1 https://doi.org/10.1080/21645515.2021.2007707	Yes	Peer	PAHO	HIC	Canada	Children	Immunizaiton	EIR	None	January to November in 2019 and 2020	Yes	Decrease with recovery		No		NA
137	Kirmani	Impact of COVID-19 pandemic on paediatric services at a referral centre in Pakistan: lessons from a low-income and middle-income country setting	Arch Dis Child 2021;106:627–628. doi:10.1136/archdischild-2020-319424	No	Peer	SEARO	LMIC	Pakistan	Children	Immunization & Child Health	Hosp IS	None	Jan-May 2020	No	Decrease		Yes	Epidemic Prev & Control	NA
138	Kofoed	The impact of COVID-19 lockdown on glycaemic control and use of health services among children followed at a Danish diabetes clinic	Acta Paediatrica. 2022;111:368–375.	No	Peer	EURO	HIC	Denmark	Children	Specialty	EMR	None	March Jan 2018-2021	Yes		StableMM	Yes	Telemedicine	Stable outcomes; Similar visits overall
139	Konson	Quality of care indicator performance was minimally changed in 2020 despite the COVID-19 pandemic	Israel Journal of Health Policy Research (2022) 11:9 https://doi.org/10.1186/s13584-022-00516-x	Yes	Peer	EMRO	HIC	Israel	MNH	MNH	HMIS	None	2019-2020	Yes	Mixed		No		NA

	A	C	D	E	F	G	H	I	J	K	M	N	O	P	S	T	U	V	Y
140	Kotiso	Impact of the COVID-19 pandemic on the utilisation of health services at public hospitals in Yemen: a retrospective comparative study	BMJ Open 2022;12:e047868. doi:10.1136/ bmjopen-2020-047868	Yes	Peer	EMRO	LMIC	Yemen	MCH	MCH	Hosp IS	None	Jan-Jun 2019-2020	No	Stable		No		NA
141	Kovler	Increased proportion of physical child abuse injuries at a level I pediatric trauma center during the Covid-19 pandemic	Child Abuse & Neglect 116 (2021) 104756	No	Peer	PAHO	HIC	USA	Children	Child Abuse	Paper MR/HR	NO Discussed limitations of diagnosis of child abuse	March-April 2018,2019,2020	No		IncreaseMM	None		NA
142	Kugelman	Consequences of the COVID-19 pandemic on the postpartum course: Lessons learnt from a large-scale comparative study in a teaching hospital	Int J Obs Gyn Volume153, Issue2 May 2021 Pages 315-321	Yes	Peer	EMRO	HIC	Israel	Maternal	Maternal Health	EMR	None	March and April 2020 (first wave), between July to September 2020 (second wave), and a matched historical cohort throughout 2017–2019	Yes	Stable	StableMM	Yes	Protocol Change	Stable Outcomes
143	Kujawski	Impact of the COVID-19 pandemic on pediatric and adolescent vaccinations and well child visits in the United States: A database analysis	Vaccine 40 (2022) 706–713	No	Peer	PAHO	HIC	USA	Children	Child Health	Admin/Billing	None	January 2018–March 2021	Yes	Decrease with Recovery		No		NA
144	Kutluk	The effect of the COVID-19 pandemic on paediatric cancer care: lessons learnt from a major paediatric oncology department in Turkey	ecancer 2021, 15:1172 https://doi.org/10.3332/ecancer.2021.1172	No	Peer	EURO	LMIC	Turkey	Children	Specialty	Hosp IS	YES do not think the decreases in the treatments weres due to treatment modifications since we only postponed the appointments for off-therapy patients, and never cancelled any ongoing treatments. However, due to uncertainity and different sources of information, the numbers of admissions, surgery, chemotherapy, radiotherapy decreased significantly as we reported in this study.	'COVID-19 period' (10 March to 31 October 2020) were compared with the corresponding 'prior year control period' (10 March to 31 October 2019)	Yes	Mixed		None		NA
145	Lamichhane Basant	TREND OF HOSPITAL CARE SERVICES DURING COVID-19 PANDEMIC IN A TERTIARY CARE HOSPITAL IN PROVINCE 5	Medphoenix. 2021;6(1):36-39 DOI:https://doi.org/10.3126/medphoenix. v6i1.36626	Yes	Peer	SEARO	LMIC	Nepal	Maternal	Maternal Health	Paper MR/HR	None	August 16th, 2019 to October 15th, 2020.	Yes	Increase		No		NA
146	Langan	Impact of the COVID-19 pandemic on radiology appointments in a tertiary children's hospital: a retrospective study	BMJ Paediatrics Open 2021;5:e001210. doi:10.1136/ bmjpo-2021-001210	No	Peer	Euro	HIC	UK	Children	Specialty	Hosp IS	None	(13 May 2019–28 March 2021	No	Mixed		None		NA
147	Langdon-Embry	Rebound in Routine Childhood Vaccine Administration Following Decline During the COVID-19 Pandemic — New York City, March 1–June 27, 2020	MMWR / July 31, 2020 / Vol. 69 / No. 30	Yes	Peer	PAHO	HIC	USA	Children	Immunization	EIR	None	December 2019–June 2020	No	Decrease with recovery		Yes	Info Campaign	Vaccination rates increased to earlier levels
148	Lee	Delay in childhood vaccinations during the COVID-19 pandemic	Canadian Journal of Public Health (2022) 113:126–134 https://doi.org/10.17269/s41997-021-00601-9	Yes	Peer	PAHO	HIC	Canada	Children	Immunizations	EMR	None	November 1, 2018 and May 31, 2020,	No	Decrease		No		NA
149	Leeb	Mental Health-Related Emergency Department Visits Among Children Aged <18 Years During the COVID-19 Pandemic - United States, January 1-October 17, 2020	MMWR Morb Mortal Wkly Rep . 2020 Nov 13;69(45):1675-1680. doi: 10.15585/mmwr.mm6945a3 .	No	Peer	PAHO	HIC	USA	Children	Mental Health	Surveillance	None	January 1 through October 17, 2020, vs 2019	Yes	Mixed		No		NA
150	Licheni	Impact of COVID-19 public health restrictions on hospital admissions for young infants in Victoria, Australia	Journal of Paediatrics and Child Health (2022) doi:10.1111/jpc.15885	Yes	Peer	WPRO	HIC	Australia	Children	EMS	Paper MR/HR	None	March to September in 2019 and 2020.	Yes	Mixed		No		NA
151	M Gorny	health in children and young people during a pandemic: experiences from North Central London during the first wave of COVID-19	BMJ Paediatrics Open 2021;5:e001116. doi:10.1136/ bmjpo-2021-001116	Yes	Peer	EURO	HIC	UK	Children	Mental Health	Hosp IS	None	Jan-May 2020	No	Increase	IncreaseMM	Yes	Relocation of services; protocls	improved quality of care

	A	C	D	E	F	G	H	I	J	K	M	N	O	P	S	T	U	V	Y
152	Maassel	Hospital Admissions for Abusive Head Trauma at Children's Hospitals During COVID-19	PEDIATRICS Volume 148, number 1, July 2021:e2021050361	No	Peer	PAHO	HIC	USA	Children	Child Abuse	Network Database	YES Major limitation is its reliance on diagnostic coding, which may be erroneous. Additionally, admission criteria may have been more restricted during COVID-19; however, this effect may be minimal, given a similar percentage of ICU admissions, compared to previous years.	2017-2020	Yes	Decrease		None		NA
153	Maeda	Trends in intensive neonatal care during the COVID-19 outbreak in Japan	Arch Dis Child Fetal Neonatal Ed 2021;106:F327–F329.	No	Peer	WPRO	HIC	Japan	Newborn	Neonatal	Hosp IS	None	10–17 vs weeks 2–9 (during and before the outbreak) of 2020 with adjustment for the trends during the same period of 2019.	No	Decrease	DecreaseMM	No		NA
154	Mahmassani	The impact of COVID-19 lockdown measures on ED visits in Lebanon	Am J Emerg Med. 2021 Aug; 46: 634–639 doi: 10.1016/j.ajem.2020.11.067	No	Peer	EMRO	LMIC	Lebanon	Children	EMS	Hosp IS	None	November 2019 to Feb 20 2020 vs Feb 21 to May 2020	No	Decrease		None		NA
155	Mann	Did children 'stay safe'? Evaluation of burns presentations to a children's emergency department during the period of COVID-19 school closures	Arch Dis Child 2021;106:e18. doi:10.1136/archdischild-2020-320015	Yes	Peer	EURO	HIC	UK	Children	EMS	Hosp IS	None	2019-2020	Yes	Mixed		No		NA
156	Markham	Inpatient Use and Outcomes at Children's Hospitals During the Early COVID-19 Pandemic	Pediatrics. 2021; 147(6):e2020044735	Yes	Peer	PAHO	HIC	USA	Children	Hosp	Network Database	NO 4 hospitals excluded for poor data quality	March 15 to August 29, 2017-2020	No	Decrease	StableMM	No		NA
157	Marques-Fernandez	Impact of Covid-19 on attendances for a 1st episode of reduced fetal movements: A retrospective observational study	PLoS ONE 16(6): e0253796. https://doi.org/10.1371/journal.pone.0253796	No	Peer	EURO	HIC	UK	MNH	MNH	EMR/EHR	None	01/03/2020-30/04/2020 (COVID) to 01/03/ 2019-30/04/2019 (Pre-COVID).	No	Decrease	StableMM	None		NA
158	Maslin	Preterm births in South-West England before and during the COVID-19 pandemic: an audit of retrospective data	European Journal of Pediatrics (2022) 181:859–863 https://doi.org/10.1007/s00431-021-04265-y	Yes	Peer	EURO	HIC	UK	Newborn	Neonatal	Registry	None	2018-2020	Yes	Decrease	StableMM	No		NA
159	Mason	Reduced presentations with fractures or orthopaedic infections to a major children's hospital during a national COVID-19 elimination strategy	ANZ J Surg 92 (2022) 206–211	Yes	Peer	WPRO	HIC	New Zealand	Children	Specialty	Hosp IS	None	March-May 2019-2020	No	Mixed		Yes		Outpatient procedures increased
160	Masresha	The performance of routine immunization in selected African countries during the first six months of the COVID-19 pandemic	Pan African Medical Journal. 2020;37(1):12. 10.11604/pamj.supp.2020.37.1.26107	No	Peer	AFRO	LMIC	Multiple	Children	Immunization	EIR	YES Completeness of district reporting of immunization data for the first 6 months of the years 2018 - 2020 was >95% in all countries except for South Sudan, which had a completeness of 93% and 96% in 2018 and 2019, while completeness for the first half of 2020 was 91%.	January 2018 to June 2020	No	Mixed		Yes	Change Service Platform	Some rebound is seen
161	Mbithi	Assessing the Real-Time Impact of COVID-19 on TB and HIV Services: The Experience and Response from Selected Health Facilities in Nairobi, Kenya	Trop. Med. Infect. Dis. 2021, 6, 74. https://doi.org/10.3390/tropicalmed6020074	No	Peer	AFRO	LMIC	Kenya	Children	HIV/TB	HMIS	YES Increased DQA during period	first six months of COVID-19 (March to August 2020) and the second six months of COVID-19 (September 2020 to February 2021)	Yes	Decrease with slight recovery		Yes	Data for targeting	TB treatment success initially decreased, it then picked up to between 70% and 80% in the last four months of the study
162	McDonald	Early impact of the coronavirus disease (COVID-19) pandemic and physical distancing measures on routine childhood vaccinations in England, January to April 2020	Euro Surveill. 2020;25(19):pii=2000848. https://doi.org/10.2807/1560-7917.ES.2020.25.19.2000848	Yes	Peer	EURO	HIC	UK	Children	Immunization	EMR/EHR	YES Deferral of data entry could explain some decrease in real-time vaccination counts, but not the subsequent increase so COVID not considered a factor in reporting	first 17 weeks of 2019 and 2020	No	Mixed		None		NA
163	McDonnell	Assessing the Impact of COVID-19 Public Health Stages on Paediatric Emergency Attendance	Int. J. Environ. Res. Public Health 2020, 17, 6719; doi:10.3390/ijerph17186719	No	Peer	Euro	HIC	Ireland	Children	EMS	EMS IS	None	Jan-May 2020 vs 2018/2019	No	Decrease		None		NA
164	McDonnell*	The impact of the Covid-19 pandemic on maternity services: A review of maternal and neonatal outcomes before, during and after the pandemic	European Journal of Obstetrics & Gynecology and Reproductive Biology 255 (2020) 172–176	Yes	Peer	EURO	HIC	Ireland	MNH	MNH	Hosp IS	None	Jan-July 2018,2019,2020	No	Stable	DecreaseMM	No		NA

	A	C	D	E	F	G	H	I	J	K	M	N	O	P	S	T	U	V	Y
165	McNicholas	Referral patterns for specialist child and adolescent mental health services in the Republic of Ireland during the COVID-19 pandemic compared with 2019 and 2018	BJPsych Open (2021) 7, e91, 1–7. doi: 10.1192/bjo.2021.48	No	Peer	EURO	HIC	Ireland	Children	EMS	HMIS	None	Jan-Nov 2019 vs same period 2020	No	Mixed		None		NA
166	McQuaid	Uptake of infant and preschool immunisations in Scotland and England during the COVID-19 pandemic: An observational study of routinely collected data	PLoS Med19(2): e1003916. https:// doi.org/10.1371/journal.pmed.1003916	Yes	Peer	EURO	HIC	UK	Chidlren	Immunizations	EIR	None	2019-Oct2020	Yes	Mixed		Yes	Outreach	Improved in Scotland compared to England
167	Meyer	Impact of the COVID-19 outbreak on routine obstetrical management: a cohort study	https://www.authorea.com/users/316659/articles/446791-impact-of-the-covid-19-outbreak-on-routine-obstetrical-management-a-cohort-study?commit=f7f2f113eb06079ac4141fe52765762b37bd737f	Yes	Pre-pub	EMRO	HIC	Israel	MNH	MNH	Paper MR/HR	None	Feb-Marc 2019 2020	No	Stable	StableMM	No		NA
168	Middeldorp	Short term impact of the COVID-19 pandemic on incidence of vaccine preventable diseases and participation in routine infant vaccinations in the Netherlands in the period March-September 2020	Vaccine 39 (2021) 1039–1043 https://doi.org/10.1016/j.vaccine.2020.12.0800264-410X	No	Peer	EURO	HIC	Netherlands	Children	Immunization	EIR	None	January 2019 through September 2020 were compared with data from the five preceding years	Yes	Decrease with recovery		Yes	Info Campaign	Recovery of vaccination rates
169	Miron	Pediatric Emergencies and Hospital Admissions in the First Six Months of the COVID-19 Pandemic in a Tertiary Children's Hospital in Romania	Children 2022, 9, 513. https:// doi.org/10.3390/children9040513	Yes	Peer	EURO	HIC	Romania	Children	EMS	Hosp IS	None	March–August 2019 2020	No	Decrease	IncreaseMM	Yes	Telemedicine	NA
170	Monroe	The Epidemic Within the Pandemic: Pediatric Mental Health Demands in the Setting of COVID-19	Clinical Pediatrics Vol 61, Issue 3, 2022 https://doi-org.ez.lshtm.ac.uk/10.1177%2F00099228211067903	Yes	Peer	PAHO	HIC	USA	Children	Mental Health	Paper MR/HR	NO Comparison of paper vs electronic MR	July 2017-Jan2021	Yes	Increase		No		NA
171	Mor	Impact of the COVID-19 Pandemic on Excess Perinatal Mortality and Morbidity in Israel	Am J Perinatol 2021;38:398–403.	No	Peer	EMRO	HIC	Israel	MNH	MNH	EMR	None	February 21 and April 30 2017-2020	No	Decrease	MixedMM	No		NA
172	Moreno-Montoya	Impact of the COVID-19 pandemic on routine childhood immunisation in Colombia	Arch Dis Child Epub ahead of print: [please include Day Month Year]. doi:10.1136/archdischild-2021-321792	Yes	Peer	PAHO	LMIC	Colombia	Children	Immunization	EIR	NO Discussed routine DQA methods, noted no precise data available on delays	March and October 2019 and during the same period in 2020;	Yes	Decrease		None		NA
173	Moura	The impact of COVID-19 on routine pediatric vaccination delivery in Brazil	Vaccine Volume 40, Issue 15, 1 April 2022, Pages 2292-2298	Yes	Peer	PAHO	LMIC	Brazil	Children	Immunizations	HMIS	None	2015-2020	Yes	Decrease		No		NA
174	Muhoza	Routine Vaccination Coverage — Worldwide, 2020	Morbidity and Mortality Weekly Report Weekly / Vol. 70 / No. 43 October 29, 2021	No	Peer	Global	Global	Global	Children	Immunizations	Surveillance	None	2010, 2019, 2020	Yes	Decrease		No		NA
175	Munaretto	Acute events in children with sickle cell disease in Italy during the COVID-19 pandemic: useful lessons learned	2021 British Society for Haematology and John Wiley & Sons Ltd doi: 10.1111/bjh.17546	No	Peer	Euro	HIC	Italy	Children	EMS	Network Database	None	2019 vs 2020 (21 February–30 April 2020)	No	Mixed		Yes	Telemedicine	Improved Health of children with SCD
176	Musser	Child maltreatment in the time of COVID-19: Changes in the Florida foster care system surrounding the COVID-19 safer-at-home order	Child Abuse & Neglect 116 (2021) 104945 https://doi.org/10.1016/j.chiabu.2021.104945	No	Peer	PAHO	HIC	USA	Children	Child Abuse	HMIS	None	April 2019 vs April 2020	No	Mixed		None		NA
177	Mutyambizi	Effect of COVID-19 on HIV, tuberculosis, and prevention of mother-to-child transmission of HIV indicators in Mopani district, South Africa	S Afr Med J 2021;111(12):1181-1189. https://doi.org/10.7196/SAMJ.2021.v111i12.15822	No	Peer	AFRO	LMIC	South Africa	MCH	HIV/TB	HMIS	None	January March 2020 and April - June 2020 periods, and differences in means for the period April - December 2019 v. the period April - December 2020	Yes	Mixed		No		NA
178	Nigeria MOH	Health Service Uptake during COVID-19 Interactive Website	https://msdat.fmohconnect.gov.ng/covid/index.html	Yes	Country Database	AFRO	LMIC	Nigeria	MCH	MCH	HMIS	YES NHMIS Reporting Rate lower during 2020 but 2021 similar to 2019	2019-2021	Yes	Mixed		None		NA

	A	C	D	E	F	G	H	I	J	K	M	N	O	P	S	T	U	V	Y
179	Nourazari	Decreased hospital admissions through emergency departments during the COVID-19 pandemic	American Journal of Emergency Medicine https://doi.org/10.1016/j.ajem.2020.11.029	Yes	Peer	PAHO	HIC	USA	Children	Hosp	Admin/Billing	None	1/1/ 2019–9/9/2019 and 1/1/2020–9/8/2020	Yes	Decrease		No		NA
180	Nuzhath	Childhood immunization during the COVID-19 pandemic in Texas	Vaccine 39 (2021) 3333–3337 https://doi.org/10.1016/j.vaccine.2021.04.050	No	Peer	PAHO	HIC	USA	Children	Immunization	EIR	None	2010-2020	Yes	Decrease		None		NA
181	Olszewska	Management of the Oral Health of Children During the COVID-19 Pandemic in Poland	Front. Public Health 9:635081. doi: 10.3389/fpubh.2021.635081	No	Peer	EURO	HIC	Poland	Children	Specialty	Paper MR/HR	NO correctly filled forms only were accepted in the study and these served as a sources of data. - No mention of rate of incorrect or incomplete forms.	March and April of 2019 compared to same period 2020	No	Mixed		None		NA
182	Oulasvirta	Paediatric prehospital emergencies and restrictions during the COVID-19 pandemic: a population-based study	BMJ Paediatrics Open 2020;4:e000808. doi:10.1136/ bmjpo-2020-000808	No	Peer	EURO	HIC	Finland	Children	EMS	EMS IS	None	March 2020–31 May 2020 (study period) and equivalent periods in 2017–2019 (control periods)	No	Mixed	IncreaseMM	None		NA
183	Özbay	The impact of Covid-19 pandemic related lockdown on clubfoot practice	Medicine (2021) 100:25	Yes	Peer	EURO	HIC	Turkey	Children	Specialty	Hosp IS	None	March-Dec 2018-2020	Yes	Increase		No		NA
184	Paiva	Impact of COVID-19 on a paediatric emergency service	European Journal of Pediatrics https://doi.org/10.1007/s00431-021-04095-y	No	Peer	EURO	HIC	Portugal	Children	EMS	Hosp IS	None	March - June 2017-2020	No	Mixed	MixedMM	None		NA
185	Park	Impact of the COVID-19 Pandemic on Pediatric Surgical Volume in Four Low- and Middle-Income Country Hospitals: Insights from an Interrupted Time Series Analysis	World J Surg (2022) 46:984–993 https://doi.org/10.1007/s00268-022-06503-2	Yes	Peer	PAHO, AFRO	LMIC	Burkina Faso, Ecuador, Nigeria, and Zambia	Children	Hosp	Network Database	None	January 1, 2019, to April 30, 2021,	Yes	Decrease	DecreaseMM	No		NA
186	Patel Murthy	Impact of the COVID-19 Pandemic on Administration of Selected Routine Childhood and Adolescent Vaccinations — 10 U.S. Jurisdictions, March–September 2020	MMWR / June 11, 2021 / Vol. 70 / No. 23	Yes	Peer	PAHO	HIC	USA	Children	Immunization	EIR	None	March–May 2020, and June– September 2020 vs 2018 and 2019	Yes	Decrease		None		NA
187	PATH	RMNCAH-N Services During COVID-19: A spotlight on India's policy responses to maintain and adapt essential health services	https://path.azureedge.net/media/documents/India_RMNCH_Deep_Dive_brief.pdf	No	Grey	SEARO	LMIC	India	MCH	MCH	HMIS	None	2018-2020	Yes	Decrease with recovery		None		NA
188	PATH	RMNCAH-N Services During COVID-19: A spotlight on Nigeria's policy responses to maintain and adapt essential health services	https://path.azureedge.net/media/documents/Nigeria_RMNCH_Deep_Dive_brief.pdf	No	Grey	AFRO	LMIC	Nigeria	MCH	MCH	HMIS	None	April 2020 compared to April 2019	No	Mixed		None		NA
189	Patkar-Kattimani	COVID-19 and obstetric anaesthetic services in a tertiary maternity care unit	International Journal of Obstetric Anesthesia (2021) 45, 152–153	Yes	Peer	EURO	HIC	UK	Maternal	Maternal Health	Hosp IS	None	October 1 to December 31, 2019 (pre COVID-19) were compared with a three-month pandemic period from March 12 to June 11, 2020 (COVID-19).	No	Stable		No		NA
190	Pelletier,	Trends in US Pediatric Hospital Admissions in 2020 Compared With the Decade Before the COVID-19 Pandemic	JAMA Network Open. 2021;4(2):e2037227. doi:10.1001/jamanetworkopen.2020.37227	No	Peer	PAHO	HIC	USA	Children	EMS	Network Database	None	2010-2020	Yes	Decrease		None		NA
191	Percul	Impact of the COVID-19 pandemic on the pediatric population with acute appendicitis: Experience at a general, tertiary care hospital	Arch Argent Pediatr 2021;119(4):224-229	Yes	Peer	PAHO	LMIC	Argentina	Children	Specialty	Hosp IS	None	March-Aug 2019 2020	No	Decrease	StableMM	Yes	Protocol Change	StableMM

	A	C	D	E	F	G	H	I	J	K	M	N	O	P	S	T	U	V	Y
192	Philip	Unprecedented reduction in births of very low birthweight (VLBW) and extremely low birthweight (ELBW) infants during the COVID-19 lockdown in Ireland: a 'natural experiment' allowing analysis of data from the prior two decades	BMJ Global Health 2020;5:e003075. doi:10.1136/ bmjgh-2020-003075	No	Peer	EURO	HIC	Ireland	Neonatal	Neonatal	Hosp IS	None	Jan-April 2001-2020	No		DecreaseMM	None		NA
193	Potenza	Business as usual during the COVID-19 pandemic? Reflections on state-wide trends in maternity telehealth consultations during lockdown in Victoria and New South Wales	ANZJOG Volume61, Issue6 December 2021 Pages 982-985 https://doi.org/10.1111/ajo.13438	Yes	Peer	WPRO	HIC	Australia	Maternal	Maternal Health	HMIS	None	1 January 2018 to 30 April 2021	Yes	Stable		Yes	Telemedicine	Maintain overall contacts by using telehealth contacts
194	Quifer-Rada,	Impact of COVID-19 Pandemic in Breastfeeding Consultations on LactApp, an m-Health Solution for Breastfeeding Support	TELEMEDICINE and e-HEALTH DOI: 10.1089/tmj.2021.0586	Yes	Peer	EURO	HIC	Spain	MNH	MNH	HMIS	None	July 2018 and March 2021	Yes	Increase		Yes	Telemedicine	Increased us of app
195	Qureshi	Impact of COVID-19 Lockdown on Admissions to a Tertiary Maternity Hospital in Srinagar	Journal of South Asian Federation of Obstetrics and Gynaecology (2021): 10.5005/jp-journals-10006-1857	Yes	Peer	SEARO	LMIC	India	Maternal	EMS	Hosp IS	None	March 2020 to August 2020 (COVID-19 data) March 2019 to August 2019 (pre-COVID-19)	No	Decrease	MixedMM	No		NA
196	R Mongru	Retrospective analysis of North West London healthcare utilisation by children during the COVID-19 pandemic	BMJ Paediatrics Open 2022;6:e001363. doi:10.1136/ bmjpo-2021-001363	Yes	Peer	EURO	HIC	UK	Children	EMS/Hosp	HMIS	None	2015 and 2021.	Yes	Decrease		No		NA
197	Radhakrishnan	Pediatric Emergency Department Visits Before and During the COVID-19 Pandemic — United States, January 2019–January 2022	MMWR / February 25, 2022 / Vol. 71 / No. 8	No	Peer	PAHO	HIC	USA	Children	EMS	Surveillance	None	2019– January 2022.	Yes	Decrease with slight recovery		No		NA
198	Rahman	Impact of the COVID-19 lockdown on routine vaccination in Pakistan: a hospital-based study	Vaccines & Immunotherapeutics, 17:12, 4934-4940, DOI: 10.1080/21645515.2021.1979380	Yes	Peer	SEARO	LMIC	Pakistan	Chidlren	Immunizations	EIR	None	01 November 2019 to the end of March, 2020 was defined as pre-lockdown 5. The period from 01 April 2020 to end of August, 2020	No	Decrease with slight recovery		No		NA
199	Raitio	Reduced number of pediatric orthopedic trauma requiring operative treatment during COVID-19 restrictions: A nationwide cohort study	Scandinavian Journal of Surgery 2021, Vol. 110(2) 254–257	Yes	Peer	EURO	HIC	Finland	Chidlren	EMS/Hosp	Hosp IS	None	March and 31 May were identified covering years from 2017 to 2020	No	Decrease	DecreaseMM	No		NA
200	Rana	Post-disruption catch-up of child immunisation and health-care services in Bangladesh	Lancet Infection March 30, 2021 https://doi.org/10.1016/ S1473-3099(21)00148-1	Yes	Peer	SEARO	LMIC	Bangladesh	Chidlren	Immunization	HMIS	None	2019 and 2020	Yes	Decrease with Recovery		Yes	Multiple	Recovery/catchup of vaccination rates
201	Rana	Emergence of measles during the COVID-19 pandemic threatens Pakistan's children and the wider region	Nature MediciNe | VOL 27 | JuLy 2021 | 1126–1134	Yes	Peer	SEARO	LMIC	Pakistan	Children	Child Health	Surveillance	None	Jan-Apr 2016-2021	Yes		IncreaseMM	No		NA
202	Ranjbar	Changes in pregnancy outcomes during the COVID-19 lockdown in Iran	BMC Pregnancy and Childbirth (2021) 21:577 https://doi.org/10.1186/s12884-021-04050-7	No	Peer	EMRO	HIC	Iran	Maternal	Mat Health	Paper MR/HR	None	pre-Covid-19 pandemic (February 19 to April 19, 2019) and the intra-Covid- 19 pandemic (February 19 to April 19, 2020) period.	No	Mixed	MixedMM	None		NA
203	Raucci	Impact of the COVID-19 pandemic on the Emergency Department of a tertiary children's hospital	Italian Journal of Pediatrics (2021) 47:21 https://doi.org/10.1186/s13052-021-00976-y	Yes	Peer	EURO	HIC	Italy	Chidlren	EMS	Hosp IS	None	Feb April 2019-2020	No	Mixed		No		NA
204	Rhedin	Reduction in paediatric emergency visits during the COVID-19 pandemic in a region with open preschools and schools	Acta Paediatrica. 2021;110:2802–2804.	Yes	Peer	EURO	HIC	Sweden	Children	EMS	Admin/Billing	None	2018-2020	Yes	Decrease		No		NA
205	Rozenfeld	COVID-19 Changed the Injury Patterns of Hospitalized Patients	Prehospital and Disaster Medicine https://doi.org/10.1017/S1049023X21000285	Yes	Peer	EMRO	HIC	Israel	Children	EMS	Registry	NO Standard data cleaning	March 15-April 30 2016-2020	No	Mixed		No		NA
206	Rusconi	Delayed presentation of children to the emergency department during the first wave of COVID-19 pandemic in Italy: Areabased cohort study	Acta Paediatrica. 2021;110:2796–2801. DOI: 10.1111/apa.16019	Yes	Peer	EURO	HIC	Italy	Children	EMS	Admin/Billing	None	Feb-May in 2018,2019, 2020	No	Decrease		None		NA

	A	C	D	E	F	G	H	I	J	K	M	N	O	P	S	T	U	V	Y
207	Sabbatucci	Childhood Immunisation Coverage during the COVID-19 Epidemic in Italy	Vaccines 2022, 10, 120. https://doi.org/ 10.3390/vaccines10010120	Yes	Peer	EURO	HIC	Italy	Chidlren	Immunizatio n	EIR	None	2019 and 2020	Yes	Mixed		No		NA
208	Sakowicz	The association between the COVID-19 pandemic and postpartum care provision	Am J Obstet Gynecol MFM 2021;3:100460. http://dx.doi.org/10.1016/j. ajogmf.2021.100460	No	Peer	PAHO	HIC	USA	Maternal	Mental Health	EMR/EHR	None	September 1, 2018, and January 1, 2019 vs February 1, 2020, and May 15, 2020	No	Decrease		None		NA
209	Salsi,	Obstetrics and gynecology emergency services during the coronavirus disease 2019 pandemic	NOVEMBER 2020 AJOG MFM	No	Peer	EURO	HIC	Italy	Maternal	EMS	Paper MR/HR	None	March 2019 vs 2020	No	Decrease	IncreaseMM	No		NA
210	Sanford	Changes in pediatric trauma during COVID-19 stay-at-home epoch at a tertiary pediatric hospital	Journal of Pediatric Surgery 56 (2021) 918–922	Yes	Peer	PAHO	HIC	USA	Children	EMS	Registry	None	March 15th thru May 15th during the years 2015–2020.	No	Mixed		No		NA
211	Sano	Large decrease in paediatric hospitalisations during the COVID-19 outbreak in Japan	BMJ Paediatrics Open 2021;5:e001013. doi:10.1136/ bmjpo-2020-001013	Yes	Peer	WPRO	HIC	Japan	Chidlren	Hosp	Admin/Billin g	None	Jan-May 2017 to 2020	No	Decrease		No		NA
212	Santoli	Effects of the COVID-19 Pandemic on Routine Pediatric Vaccine Ordering and Administration — United States, 2020	MMWR / May 15, 2020 / Vol. 69 / No. 19	No	Peer	PAHO	HIC	USA	Children	Immunizatio n	EIR, LMIS	None	Jan to April 2020	No	Decrease		None		NA
213	Saunders	Pediatric primary care in Ontario and Manitoba after the onset of the COVID-19 pandemic: a population-based study	CMAJ OPEN, 9(4) E1149-1158	Yes	Peer	PAHO	HIC	Canada	Chidlren	Child Health	Admin/Billin g	None	Jan. 1, 2017, and Nov. 28, 2020	Yes	Decrease with slight recovery		No		NA
214	Selinger	Impact of the coronavirus infectious disease (COVID-19) pandemic on the provision of inflammatory bowel disease (IBD) antenatal care and outcomes of pregnancies in women with IBD	BMJ Open Gastro 2021;8:e000603. doi:10.1136/ bmjgast-2021-000603	No	Peer	EURO	HIC	UK	Maternal	Maternal Health	Paper MR/HR	NO Mostly retrospective data collection has led to some missing data (not quantified), which may introduce bias	March to August 2020 compared to unspecified pre-pandemic period	No	Stable		Yes	Telemedicine	Stated outcomes reassuring but no comparison included, only descriptive stats
215	Semaan	'We are not going to shut down, because we cannot postpone pregnancy': a mixed-methods study of the provision of maternal healthcare in six referral maternity wards in four sub-Saharan African countries during the COVID-19 pandemic	BMJ Global Health 2022;7:e008063. doi:10.1136/ bmjgh-2021-008063	Yes	Peer	AFRO	LMIC	Guinea, Nigeria, Tanzania, Uganda	Maternal	Maternal Health	Paper HMIS	None	1 January 2019 and 28 February 2021	Yes	Stable		Yes	Multiple	Stable Access
216	Sen	Disparities in Telehealth Utilization in a Population of Publicly Insured Children During the COVID-19 Pandemic	Population Health Management.Apr 2022.178-185.http://doi.org/10.1089/ pop.2021.0343 Published in Volume: 25 Issue 2: April 19, 2022	Yes	Peer	PAHO	HIC	USA	Chidlren	Child Health	Admin/Billin g	None	March-Dec 2019 vs 2020	Yes	Mixed		Yes	Telemedicine	Increase telemed
217	Sevalie	The impact of the COVID-19 pandemic on hospital utilisation in Sierra Leone	BMJ Global Health 2021;6:e005988. doi:10.1136/ bmjgh-2021-005988	Yes	Peer	AFRO	LMIC	Sierra Leone	MCH	MCH	Hosp IS	None	1 January 2020 to 30 September 2020	Yes	Stable		No		NA

	A	C	D	E	F	G	H	I	J	K	M	N	O	P	S	T	U	V	Y
218	Shakespeare	Resilience and vulnerability of maternity services in Zimbabwe: a comparative analysis of the efect of Covid-19 and lockdown control measures on maternal and perinatal outcomes, a single-centre cross-sectional study at Mpilo Central Hospital	BMC Pregnancy Childbirth (2021) 21:416 https://doi.org/10.1186/s12884-021-03884-5	No	Peer	AFRO	LMIC	Zimbabwe	MNH	MNH	Hosp IS	YES A major strength of this study is that it relies on routinely collected and contemporaneously reported data from the maternity department. This means that data is readily and quickly available to monitor outcomes and trends, and any systematic errors in data collection are likely to remain constant from month to month. During the study period there was no statistical diference in the number of man-hours worked before and during the lockdown, indicating that the unit received the usual personnel coverage, hence there was little changes in clinical outcomes. However, it is possible that if wards were to be extremely short-staffed and staff working under additional pressure due to the pandemic, this may afect the way they report outcomes,	Jan-March versus April-June 2020	No	Decrease	StableMM	None		NA
219	Shalitin	Changes in body mass index in children and adolescents in Israel during the COVID-19 pandemic	International Journal of Obesity; https://doi.org/10.1038/s41366-022-01092-5	Yes	Peer	EMRO	HIC	Israel	Chidlren	Child Health	EMR	None	2017–2019 (pre-pandemic period) and one between April 1, 2020 and December 31, 2020 (pandemic period).	Yes		IncreaseMM	No		NA
220	Shanmugavadivel	Changing patterns of emergency paediatric presentations during the first wave of COVID-19: learning for the second wave from a UK tertiary emergency department	BMJ Paediatrics Open 2021;5:e000967. doi:10.1136/ bmjpo-2020-000967	Yes	Peer	EURO	HIC	UK	Children	EMS	EMR	None	2 March 2020–3 May 2020) compared with 2019	No	Decrease	StableMM	No		NA
221	Shapira	Disruptions in maternal and child health service utilization during COVID-19: analysis from eight sub-Saharan African countries	Health Policy and Planning, 36, 2021, 1140–1151 DOI: https://doi.org/10.1093/heapol/czab064 Advance access publication date: 19 June 2021	Yes	Peer	AFRO	LMIC	Uganda	MCH	MCH	HMIS	YES Cite limitations of routine data, including potential reporting disruption due to COVID. Adjust for outliers and missing reports. Report data completeness in supp materials	Jan 2018 to Feb 2020	No	Decrease		None		NA
222	Sharma	COVID-19: Differences in sentinel injury and child abuse reporting	Child Abuse Negl. 2021 Jun; 116: 104990. Published	No	Peer	PAHO	HIC	USA	Children	Child Abuse	EMR/EHR	None	March 15 to July 31 of 2017, 2018, 2019, and	No	Mixed		None		NA
223	Sharma,	The Impact of COVID-19 Pandemic on Access to Treatment for Children With Cancer in India and Treating Center Practices	Cancer February 1, 2022 DOI: 10.1002/cncr.33945,	Yes	Peer	SEARO	LMIC	India	Chidlren	Specialty	Hosp IS	None	January 1, 2020, to May 31, 2020	No	Decrease		Yes	Multiple	NA
224	Sheridan	Where have all the emergency paediatric mental health patients gone during COVID-19?	Acta Paediatrica. 2021;110:598–599.	Yes	Peer	PAHO	HIC	USA	Children	EMS	EMR	None	April 1, 2019, to April 29, 2020	No	Decrease		No		NA
225	Sherman	How Did the Number and Type of Injuries in Patients Presenting to a Regional Level I Trauma Center Change During the COVID-19 Pandemic with a Stay-at-home Order?	Clinical Orthopaedics and Related Research: February 2021 - Volume 479 - Issue 2 - p 266-275 doi: 10.1097/CORR.0000000000001484	Yes	Peer	PAHO	HIC	USA	Children	EMS	Registry	None	2017 to 2020	Yes	Decrease		No		NA
226	Shichijo	Patient attendance at apediatric emergency referral hospital in an area with low COVID-19 incidence	PLoS ONE16(10): e0258478. https://doi.org/ 10.1371/journal.pone.0258478	Yes	Peer	WPRO	HIC	Japan	Children	EMS	Paper MR/HR	None	017–2019, before the COVID-19 pandemic, with 2020	Yes	Mixed		No		NA
227	Shikuku	Early indirect impact of COVID-19 pandemic on utilization and outcomes of reproductive, maternal, newborn, child and adolescent health services in Kenya	medRxiv preprint doi: https://doi.org/10.1101/2020.09.09.20191247; t	No	Pre-pub	AFRO	LMIC	Kenya	MCH	MCH	HMIS	NO However, the use of DHIS2 data poses key data quality challenges including inaccurate and incomplete reporting that are prevalent in low and middle-income countries	March to June 2019 vs 2020	No	Mixed	MixedMM	None		NA
228	Shimels	he Trend of Health Service Utilization and Challenges Faced During the COVID-19 Pandemic at Primary Units in Addis Ababa: A Mixed-Methods Study	Health Services Research and Managerial Epidemiology Volume 8: 1-8 2021 DOI: 10.1177/23333928211031119	Yes	Peer	AFRO	LMIC	Ethiopia	MCH	MCH	HMIS	None	September 2019 to July 2020	No	Stable		No		NA
229	Shimels	The Trend of Health Service Utilization and Challenges Faced During the COVID-19 Pandemic at Primary Units in Addis Ababa: A Mixed-Methods Study	Health Services Research and Managerial Epidemiology Volume 8: 1-8 DOI: 10.1177/23333928211031119	No	Peer	AFRO	LMIC	Ethiopia	MCH	MCH	HMIS	None	Sept 2019-July 2020	No	Mixed		None		NA

	A	C	D	E	F	G	H	I	J	K	M	N	O	P	S	T	U	V	Y
230	Shrinivasan	India's syndemic of tuberculosis and COVID-19	BMJ Global Health 2020;5:e003979. doi:10.1136/ bmjgh-2020-003979	No	Peer	SEARO	LMIC	India	Children	Immunization	HMIS	YES From March 2020 NHM-HMIS reported no new data for the next 3 months. In late August, data for April, May and June finally became available	Jan-June 2019 vs 2020	No	Decrease		None		NA
231	Shuka	Use of healthcare services during the COVID-19 pandemic in urban Ethiopia: evidence from retrospective health facility survey data	BMJ Open 2022;12:e056745. doi:10.1136/ bmjopen-2021-056745	No	Peer	AFRO	LMIC	Ethiopia	MCH	MCH	HMIS	None	March to June 2019 vs 2020	No	Stable		No		NA
232	Siddiqi	Using a low-cost, real-time electronic immunization registry in Pakistan to demonstrate utility of data for immunization programs and evidence-based decision making to achieve SDG-3: Insights from analysis of Big Data on vaccines	International Journal of Medical Informatics Volume 149, May 2021, 104413	No	Peer	SEARO	LMIC	Pakistan	Children	Immunizations	EIR	None	Feb-April 2020	No	Decrease		No		NA
233	Silvagni	Neonatal and Pediatric Emergency Room Visits in a Tertiary Center during the COVID-19 Pandemic in Italy	Pediatr. Rep. 2021, 13, 168–176. http://doi.org/10.3390/ pediatric13020023	No	Peer	EURO	HIC	Italy	Children	EMS	Hosp IS	None	March–April 2020 (COVID-19) and March–April 2019 (non-COVID-19) w	No	Mixed		None		NA
234	Silveira	Missed childhood immunizations during the COVID-19 pandemic in Brazil: analyses of routine statistics and of a national household survey	medRxiv preprint doi: https://doi.org/10.1101/2020.11.30.20240911;	No	Pre-Pub	PAHO	LMIC	Brazil	Children	Immunization	EIR	NO Coverage of the national information system is high, but there are delays in reporting from primary care facilities (not discussed in relation to COVID - standard delays)	2017-19 vs 2020	Yes	Decrease with recovery		None		NA
235	Simon-Tov	An assessment of treatment, transport, and refusal incidence in a National EMS's routine work during COVID- 19	American Journal of Emergency Medicine 44 (2021) 45–49 https://doi.org/10.1016/j.ajem.2021.01.051 0735-6757/	Yes	Peer	EMRO	HIC	Israel	Maternal	EMS	EMS IS	None	2019–2020 March/April periods	No	Decrease		Yes	Telemedicine	NA
236	Singh	Impact of COVID-19 pandemic on maternal and child health services in Uttar Pradesh, India	J Family Med Prim Care. 2021 Jan; 10(1): 509–513. doi: 10.4103/jfmpc.jfmpc_1550_20: 10.4103/jfmpc.jfmpc_1550_20	Yes	Peer	SEARO	LMIC	India	MCH	MCH	Paper HMIS	None	March 2019 vs 2020	No	Decrease		None		NA
237	Sokolof	Pediatric emergency department utilization during the COVID-19 pandemic in New York City	American Journal of Emergency Medicine 45 (2021) 100–104 https://doi.org/10.1016/j.ajem.2021.02.029 0735-6757	No	Peer	PAHO	HIC	USA	Children	EMS	Hosp IS	NO Common limitations of a retrospective review including dependence on data collection systems that may have missing or incorrect information.	March 7th to May 6th 2020, and during the same time period in 2018 and 2019	No	Mixed		None		NA
238	Šokota	Pediatric tertiary emergency care departments in Zagreb, Rijeka, and Split before and during the coronavirus disease 2019 pandemic: a Croatian national multicenter study	Croat Med J. 2021;62:580-9 https://doi.org/10.3325/cmj.2021.62.580	Yes	Peer	EURO	HIC	Croatia	Children	EMS	EMR	None	February 25-April 25, 2018 and 2019 (preCOVID years), and February 25-April 24, 2020	No	Decrease		No		NA
239	Solo-Josephson	Patient and Visit Characteristics of Families Accessing PediatricUrgent Care Telemedicine During the COVID-19 Pandemic	ELEMEDICINE and e-HEALTH®MARY ANN LIEBERT, INC. VOL. 28 NO. 4 APRIL 2022DOI: 10.1089/tmj.2021.0135	Yes	Peer	PAHO	HIC	USA	Children	EMS	HMIS	None	January to May 2019, 2020	No	Increase		Yes	Telemedicine	NA
240	Soma, Pattinson	The eff ect of the fi rst wave of Covid-19 on use of maternal and reproductive health services and maternal deaths in South Africa	O&G Forum 2020; 30: 36 - 44	No	Peer	AFRO	LMIC	South Africa	Maternal	Mat Health	HMIS	None	Q1-3 2019 vs Q1-3 2020	Yes	Decrease	IncreaseMM	None		NA

	A	C	D	E	F	G	H	I	J	K	M	N	O	P	S	T	U	V	Y
241	Stout	Use of Electronic Medical Records to Estimate Changes in Pregnancy and Birth Rates During the COVID-19 Pandemic	JAMA Network Open. 2021;4(6):e2111621. doi:10.1001/jamanetworkopen.2021.11621 (Reprinted) June 3, 2021 1/10	No	Peer	PAHO	HIC	USA	Maternal	Mat Health	EMR	None	a Prelockdown period was January 1, 2020, to March 28, 2020. b Postlockdown period was March 29, 2020, to June 14, 2020.	No	Decrease		None		NA
242	Stowe	Stillbirths During the COVID-19 Pandemic in England, April-June 2020	JAMA January 5, 2021 Volume 325, Number 1 (R	Yes	Peer	EURO	HIC	UK	Newborn	Stillbirth	Hosp IS	None	April 1, 2019, to June 30, 2020	No	Stable		No		NA
243	SUGAND	Impact of the COVID-19 pandemic on paediatric orthopaedic trauma workload in central London: a multi-centre longitudinal observational study over the "golden weeks"	Acta Orthopaedica 2020; 91 (6): 633–638	No	Peer	Euro	HIC	UK	Children	EMS	EMS IS	None	March 24) to the April 28, 2020 vs same in 2019	No	Decrease		None		NA
244	Sun	Effect of delayed obstetric labor care during the COVID-19 pandemic on perinatal outcomes	Int J Gynecol Obstet 2020; 151: 287–307	Yes	Peer	PAHO	LMIC	Brazil	MNH	MNH	EMR	None	March11andJune11, 2019andMarch11andJune11,2020	No	Decrease	StableMM	No		NA
245	Taquechel	Pediatric Asthma Health Care Utilization, Viral Testing, and Air Pollution Changes During the COVID-19 Pandemic	J ALLERGY CLIN IMMUNOL PRACT VOLUME 8, NUMBER 10	No	Peer	PAHO	HIC	USA	Children	Specialty	Hosp IS	NO Electronic health recordederived data is subject to bias and error more broadly, which we were unable to control for, although most errors in the data would have biased us toward not observing significant changes	January 17 to May 17, 2015 vs same period 2020	No	Decrease	DecreaseMM	Yes	Telemedicine	Possible reduced disease due to PH measures and decreased RSV
246	Thekkur	Operational Research to Assess the Real-Time Impact of COVID-19 on TB and HIV Services: The Experience and Response from Health Facilities in Harare, Zimbabwe	Trop. Med. Infect. Dis. 2021, 6, 94. https://doi.org/10.3390/tropicalmed6020094	Yes	Peer	AFRO	LMIC	Zimbabwe	Children	HIV/TB	HMIS	None	March 2019 and February 2021; March 2020 to February 2021 was the COVID-19 period, and March 2019 to February 2020 was the pre-COVID-19 period.	Yes	Decrease		Yes	Data for targeting	Unable to reverse decreases
247	Thekkur	Assessing the Impact of COVID-19 on TB and HIV Programme Services in Selected Health Facilities in Lilongwe, Malawi: Operational Research in Real Time	Trop. Med. Infect. Dis. 2021, 6, 81. https://doi.org/10.3390/tropicalmed6020081	Yes	Peer	AFRO	LMIC	Malawi	Children	HIV/TB	HMIS	None	March 2019-Feb2020 vs March 2020-Feb2021	Yes	Decrease		None		NA
248	Todd	Changes in infection-related hospitalizations in children following pandemic restrictions: an interrupted time-series analysis of total population data	International Journal of Epidemiology, 2021, 1435–1443 doi: 10.1093/ije/dyab101	Yes	Peer	WPRO	HIC	Australia	Children	Hosp	Admin/Billing	None	1 January 2015 to 3 October 2020	Yes	Decrease	DecreaseMM	No		NA
249	Uganda MOH	The effects of the COVID-19 pandemic on the continuity of essential health services delivery, access, and uptake in Uganda AUGUST 2020 Immunization, Gender-based Violence, Maternal Newborn and Child Health, Nutrition, Tuberculosis	http://library.health.go.ug/publications/disease-survielance-outbreaks/effect-covid-19-pandemic-continuity-essential-health	Yes	Country Database	AFRO	LMIC	Uganda	MCH	MCH	HMIS	YES % of facilities report 105:1 form 95%. Reporting rates on an indicator are low due to challenges, unstable internet connections, low accessibility to 105:1 form. Also affects data quality. observe unexpected trends across all Nutrition indicators, numbers can double or more in just one-month time. could be due to issues related to HMIS data entry. low reporting rates (62%, 9%, 23%) are also problem for nutrition indicators. Data entry. The data in DHIS2 is collected by several individuals, who can easily make mistakes when entering data. numbers might be affected by low reporting rates, late reporting, or wrong data entry in order to support better data-driven decision-making, health facilities need additional support to improve indicator reporting on all indicators in a timely and correct way.	Jan-Aug 2018/2019 vs 2020	No	Mixed	MixedMM	None		NA

	A	C	D	E	F	G	H	I	J	K	M	N	O	P	S	T	U	V	Y
250	Utria	Impact of COVID-19 on procedure volume at a tertiary pediatric hospital	The American Journal of Surgery 221 (2021) 1259e1261 https://doi.org/10.1016/j.amjsurg.2021.03.0030002-9610	No	Peer	PAHO	HIC	USA	Children	Specialty	EMR/EHR	YES Limited by the lack of data on 1) cancelled procedures secondary to the elective nature of the procedure or a positive COVID-19 result	March 19th, 2020 to May 18th, 2020, or during the same period during the year prior, March 19th, 2019 to May 18th, 2019	No	Mixed		Yes	Epidemic Prev & Control	COVID tests none positive
251	Velasco-Arnaiz	Pediatric antimicrobial stewardship in the COVID-19 outbreak	Infection Control & Hospital Epidemiology, 42: 642–644, https:// doi.org/10.1017/ice.2020.312	Yes	Peer	EURO	HIC	Spain	Children	Specialty	Surveillance	None	March-Apr 2019 vs 2020	No	Mixed		No		NA
252	Vogel	COVID-19 pandemic and families' utilization of well-child clinics and pediatric practices attendance in Germany	BMC Res Notes (2021) 14:140 https://doi.org/10.1186/s13104-021-05562-3	Yes	Peer	EURO	HIC	Germany	Children	Child Health	Network Database	None	March and April in 2020 to the number of visits in 2019,	No	Decrease with slight Recovery		No		NA
253	Walker	COVID-19 and Routine Childhood and Adolescent Immunizations: Evidence from Louisiana Medicaid	Vaccine Volume 40, Issue 6, 7 February 2022, Pages 837-840	No	Peer	PAHO	HIC	USA	Children	Immunization	Admin/Billing	None	January 2017 through December 2020.	Yes	Decrease		No		NA
254	Wambua	Quantifying the indirect impact of COVID-19 pandemic on utilisation of outpatient and immunisation services in Kenya: a longitudinal study using interrupted time series analysis	BMJ Open 2022;12:e055815. doi:10.1136/ bmjopen-2021-055815	Yes	Peer	AFRO	LMIC	Kenya	MCH	MCH	HMIS	NO Standard data cleaning plus missing value imputation	January 2018 to March 2021	No	Decrease with recovery		Yes	Relocation of services	where health facilities designated as vaccination centres were assigned as COVID-19 isolation centres, the vaccines programme moved immunisation services to neighbouring health facilities
255	Wang	Maternal and infant outcomes during the COVID-19 pandemic: a retrospective study in Guangzhou, China	Reprod Biol Endocrinol (2021) 19:126 https://doi.org/10.1186/s12958-021-00807-z	No	Peer	WPRO	LMIC	China	MNH	MNH	Paper MR/HR	None	24 January to 31 March 2020 (peak period), chose the same types of data at the hospital during the same period in 2019 and 1 January—23 January 2020	No	Mixed		Yes	Epidemic Prev & Control	Stable outcomes
256	Wanyana	Rapid assessment on the utilization of maternal and child health services during COVID-19 in Rwanda	PHA 2021; 11(1): 12–21 e-ISSN 2220-8372 https://doi.org/10.5588/pha.20.0057	No	Peer	AFRO	LMIC	Rwanda	MCH	MCH	HMIS	None	March and April 2019 (before the COVID-19 outbreak) were compared with those from March and April 2020	No	Mixed		None		NA
257	Ward	Describing Changes in Telebehavioral Health Utilization and Services Delivery in Rural School Settings in Pre- and Early Stages of the COVID-19 Public Health Emergency	J Sch Health. 2022; 92: 452-460. DOI: 10.1111/josh.13150	No	Peer	PAHO	HIC	USA	Children	Child Health	Network Database	None	Fall 2019 vs spring 2020	No	Increase		Yes	Telemedicine	NA
258	Weiqin Liu	Impact of the COVID-19 pandemic on neonatal admissions in atertiary children's hospital in southwest China: An interrupted time-series study	PLoS ONE17(1): e0262202. https:// doi.org/10.1371/journal.pone.0262202	Yes	Peer	WPRO	LMIC	China	Newborn	Neonatal	Paper MR/HR	None	January 4,2019, and August 27, 2020	No	Decrease	IncreaseMM	No		NA
259	Williams	Indirect effects of the COVID-19 pandemic on paediatric healthcare use and severe disease: a retrospective national cohort study	Arch Dis Child 2021;106:911–917	No	Peer	EURO	LMIC	UK	Children	EMS	HMIS	None	2016-2020	Yes	Decrease	StableMM	No		NA
260	Wong	Disruption of paediatric orthopaedic hospital services due to the COVID-19 pandemic in a region with minimal COVID-19 illness	J Child Orthop 2020;14:245-251. DOI: 10.1302/18632548.14.200140	Yes	Peer	WPRO	HIC	Australia	Chidlren	Specialty	Hosp IS	None	2019-2020	Yes	Decrease		No		NA
261	Workicho	Essential Health and Nutrition Service Provision during the COVID-19 Pandemic: Lessons from Select Ethiopian Woredas	Curr Dev Nutr 2021;5:nzab024	Yes	Peer	AFRO	LMIC	Ethiopia	Children	Child Health	Paper HMIS	None	March to July of 2019 and 2020,	No	Decrease with Recoviery		Yes	Multiple	Recovery of services
262	Yamada	Do Not Delay: Safe Operation for Pediatric Living-donor Liver Transplantation Programs in the COVID-19 Era	Transplantation: March 2021 - Volume 105 - Issue 3 - p e39-e40 doi: 10.1097/TP.0000000000003594	Yes	Peer	WPRO	HIC	Japan	Children	Specialty	Surveillance	None	Jan-Oct 2019 vs 2020	Yes	Decrease with Recovery	IncreaseMM	No		NA

	A	C	D	E	F	G	H	I	J	K	M	N	O	P	S	T	U	V	Y
263	Zacay	Decreases in pediatric fractures during the COVID-19 pandemic — a nationwide epidemiological cohort study	European Journal of Pediatrics (2022) 181:1473–1480 https://doi.org/10.1007/s00431-021-04323-5	Yes	Peer	EMRO	HIC	Israel	Children	EMS	Network Database	None	2015–2020	Yes		DecreaseMM	No		NA
264	Zarasvand	Rapid quality improvement in a preterm birth clinic care pathway during the COVID-19 pandemic	BMJ Open Quality 2020;9:e001049. doi:10.1136/ bmjoq-2020-001049	No	Peer	EURO	HIC	UK	Maternal	Mat Health	EMR/EHR	None	10-week period prior to COVID-19 between 25 November 2019 and 27 January 2020 vs 10 weeks after 27 January 2020; comparison to 3 year average prior to COVID	No	Mixed	StableMM	Yes	Telemedicine	cannot demonstrate with absolute certainty that the new care pathway did not result in an increase in potentially preventable MTLs or PTBs.
265	Zee-Cheng	Changes in Pediatric ICU Utilization and Clinical Trends During the Coronavirus Pandemic	[160#2 CHEST AUGUST 2021] DOI: https://doi.org/10.1016/j.chest.2021.03.004	No	Peer	PAHO	HIC	USA	Children	EMS	Network Database	None	quarters 1 (Q1) and 2 (Q2) of 2017 to 2019 (pre-COVID-19) and 2020 (COVID-19).	No	Decrease	IncreaseMM	None		NA
266	Zhai	An internet-based multidisciplinary online medical consultation system to help cope with pediatric medical needs during the COVID-19 outbreak: a cross-sectional study	Transl Pediatr 2021;10(3):560-568 | http://dx.doi.org/10.21037/tp-20-348	No	Peer	WPRO	LMIC	China	Children	EMS	Hosp IS	None	February 8 to March 31 2019 vs 2020	No	Decrease		Yes	Telemedicine	Use of on-line increased over time and crossed all specialties and ages
267	Zhong	Childhood vaccinations: Hidden impact of COVID-19 on children in Singapore	Vaccine Volume 39, Issue 5, 29 January 2021, Pages 780-785 https://doi.org/10.1016/j.vaccine.2020.12.054	No	Peer	SEARO	HIC	Singapore	Children	Immunization	EIR	None	Jan-March 2019 vs Jan-March 2020 & April 2019 vs April 2020	No	Decrease		None		NA
268																			
269																			
270																			
271																			
272																			
273																			
274																			
275																			
276																			
277																			
278																			
279																			
280																			
281																			
282																			
283																			
284																			
285																			
286																			
287																			
288																			
289																			
290																			
291																			
292																			
293																			
294																			
295																			
296																			
297																			
298																			
299																			
300																			
301																			
302																			
303																			
304																			
